# Buffer‐Less Gallium Nitride High Electron Mobility Heterostructures on Silicon

**DOI:** 10.1002/adma.202413127

**Published:** 2025-01-23

**Authors:** Saptarsi Ghosh, Martin Frentrup, Alexander M. Hinz, James W. Pomeroy, Daniel Field, David J. Wallis, Martin Kuball, Rachel A. Oliver

**Affiliations:** ^1^ Department of Materials Science and Metallurgy University of Cambridge Cambridge CB3 0FS UK; ^2^ Department of Electronic and Electrical Engineering Swansea University Swansea SA1 8EN UK; ^3^ H.H. Wills Physics Laboratory University of Bristol Bristol BS8 1TL UK; ^4^ Centre for High Frequency Engineering Cardiff University Cardiff CF24 3AA UK

**Keywords:** AlGaN/GaN HEMTs, GaN‐on‐Si, heteroepitaxy, magneto‐transport, thermal resistance

## Abstract

Thick metamorphic buffers are considered indispensable for III‐V semiconductor heteroepitaxy on large lattice and thermal‐expansion mismatched silicon substrates. However, III‐nitride buffers in conventional GaN‐on‐Si high electron mobility transistors (HEMT) impose a substantial thermal resistance, deteriorating device efficiency and lifetime by throttling heat extraction. To circumvent this, a systematic methodology for the direct growth of GaN after the AlN nucleation layer on six‐inch silicon substrates is demonstrated using metal‐organic vapor phase epitaxy (MOVPE). Crucial growth‐stress modulation to prevent epilayer cracking is achieved even without buffers, and threading dislocation densities comparable to those in buffered structures are realized. The buffer‐less design yields a GaN‐to‐substrate thermal resistance of (11 ± 4) m^2^ K GW^−1^, an order of magnitude reduction over conventional GaN‐on‐Si and one of the lowest on any non‐native substrate. As‐grown AlGaN/AlN/GaN heterojunctions on this template show a high‐quality 2D electron gas (2DEG) whose room‐temperature Hall‐effect mobility exceeds 2000 cm^2^ V^−1^ s^−1^, rivaling the best‐reported values. As further validation, the low‐temperature magnetoresistance of this 2DEG shows clear Shubnikov‐de‐Haas oscillations, a quantum lifetime > 0.180 ps, and tell‐tale signatures of spin‐splitting. These results could establish a new platform for III‐nitrides, potentially enhancing the energy efficiency of power transistors and enabling fundamental investigations into electron dynamics in quasi‐2D wide‐bandgap systems.

## Graphical abstract

This study presents a systematic method for the direct growth of GaN on six‐inch silicon substrates without buffers, significantly reducing GaN‐to‐substrate thermal resistance while maintaining structural quality comparable to buffered approaches. As‐grown AlGaN/AlN/GaN heterojunctions on this buffer‐less platform demonstrate state‐of‐the‐art 2D electron gas mobilities, paving the way for efficient III‐nitride transistors and advancing fundamental research on electron dynamics.

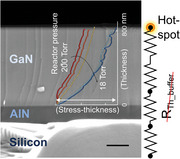

## 1 Introduction

The success of semiconductor devices behind the quantum revolution is intricately related to long‐term developments in epitaxial techniques including the control of material defects. For example, buffer optimization^[^ ^1^ ^]^ and acceptor activation^[^ ^2^ ^]^ were pivotal for the growth of InGaN/GaN quantum well (QW) based light‐emitting diodes (LEDs) by metal‐organic vapor phase epitaxy (MOVPE) and ushered in the era of energy‐efficient lighting.^[^ ^3^ ^]^ From the same III‐nitride family, heterostructures of GaN and its alloys with AlN are now a frontrunner for high‐frequency, high‐power microelectronics by harnessing these materials’ wide bandgap (3.4–6.0 eV), large breakdown field (>3 MV cm^−1^), high electron saturation velocity (≥3 × 10^7^ cm s^−1^) along with robust thermal and chemical stability. At the AlGaN/GaN interface in metal‐polar heterostructures, the combination of a lack of inversion symmetry along [0001] and a strong polarity of the metal‐nitrogen bonds leads to a polarization‐induced highly mobile 2D electron gas^[^ ^4^ ^]^ (2DEG). Its carrier density is an order of magnitude higher than modulation‐doped AlGaAs/GaAs 2DEGs, and its conductivity is superior to electron channels in competing wide bandgap SiC, Ga_2_O_3_, and diamond materials.^[^ ^5^ ^]^ High electron mobility transistors (HEMT) based on such dopant‐free 2DEGs in III‐nitrides underpin 5G radio‐frequency (RF) amplifiers,^[^ ^6^ ^]^ power converters,^[^ ^7^ ^]^ extreme environment electronics,^[^ ^8^ ^]^ chemical sensors,^[^ ^9^ ^]^ terahertz detectors,^[^ ^10^ ^]^ thermoelectric harvesters,^[^ ^11^ ^]^ and flexible electronics^[^ ^12^ ^]^ applications.

However, with one of the highest heat fluxes^[^ ^13^ ^]^ for solid‐state transistors, a major roadblock for high power density GaN HEMT circuits is the extraction of heat. High operating temperatures from localized Joule heating in the gate‐drain region not only degrade transconductance but also exponentially increase the probability of device failure.^[^ ^14^ ^]^ Hence, all thermal resistances between the channel and heat‐sink need minimization. This is particularly disadvantageous in terms of the application of thermally‐resistive sapphire (thermal conductivity, *k* ≈ 40 W m^−1^ K^−1^), the typical manufacturing platform for nitride LEDs, as a substrate for heteroepitaxial HEMTs. Instead, hexagonal SiC (*k* ≈ 400 W m^−1^ K^−1^) is considered the optimal substrate for GaN RF HEMTs as economical bulk nitride wafers are still unavailable. AlN is the commonly used nucleation layer (NL) for nitride epitaxy on non‐native substrates such as sapphire,^[^ ^15^ ^]^ SiC,^[^ ^16^ ^]^ and Si.^[^ ^17^ ^]^ SiC possess only ≈1% basal‐plane lattice parameter mismatch with AlN. Hence, for SiC, low dislocation‐density GaN on an AlN NL can be achieved without needing additional defect‐filtering layers, and this design keeps the channel‐to‐substrate aggregate thermal resistance low.^[^ ^18^ ^]^ Nonetheless, high costs and the existence of export restrictions of large‐area SiC wafers have long‐prompted the urge for their replacement with orders of magnitude cheaper large‐area silicon (*k* ≈ 150 W m^−1^ K^−1^) substrates. The latter can also leverage widely‐available technological know‐how on Si‐based processing and legacy complementary metal–oxide–semiconductor (CMOS) fabrication facilities.

Lithography‐free epitaxial integration of III‐V semiconductors on silicon wafers has traditionally relied upon buffers between the functional layers and the substrate.^[^ ^19^, ^20^ ^]^ Specifically, GaN grown on Si experiences significant tensile stress during post‐growth cooldown owing to the ≈50% mismatch in coefficients of thermal expansion (CTE) between substrate and epilayer, often cracking the epilayers. In addition, the ≈19% lattice mismatch between AlN and Si generates significantly more threading dislocations (TDs) in the NL, which in‐turn propagate into the GaN. To tackle these issues, thick compositionally graded‐AlGaN^[^ ^17^, ^21^ ^]^ or AlN/GaN superlattice layers^[^ ^22^ ^]^ on top of the NL have been integral components of GaN‐on‐Si HEMTs for the last two decades. These buffers induce epitaxial compressive stress in GaN to balance the post‐growth tensile stress and partially annihilate TDs generated in the NLs.^[^ ^21^, ^22^ ^]^ On the flip side, such nitride buffers prolong the overall growth duration, increasing the thermal budget and the usage of unsustainably‐produced ammonia and hydrogen carrier gas. This is contrary to the objectives of greener manufacturing as energy, financial, and environmental costs of semiconductor production are becoming a major concern.^[^ ^23^ ^]^ More importantly, heat‐carrying phonons suffer significant alloy scattering in Al_x_Ga_1−x_N (for 0.1 < *x* < 0.9) and interface scattering in short‐period AlN/GaN superlattices. Both of these buffers possess very‐low thermal conductivity (*k* ≈ 10 W m^−1^ K^−1^)^[^ ^24^, ^25^ ^]^ compared to either AlN^[^ ^26^ ^]^ (*k* ≈ 340 W m^−1^ K^−1^) or GaN^[^ ^27^ ^]^ (*k* ≈ 140 W m^−1^ K^−1^) and appear as large thermal resistance in series. This proportionately raises the device temperature,^[^ ^28^ ^]^ causing operational and reliability concerns. In fact, even after substrate thinning, self‐heating still lowers the RF output of HEMTs on Si at higher powers.^[^ ^29^ ^]^ Compared to benchmark results on SiC,^[^ ^30^ ^]^ today's commercial GaN‐on‐Si devices are intentionally derated by an order of magnitude. Thus, bypassing the buffer bottleneck without sacrificing the stress‐management and structural benefits they enable has become an immediate need to unleash the potential of nitride HEMTs.

With or without a buffer, offsetting the post‐growth tensile stress (*σ* ≈ 1 GPa for ≈1000 °C growth temperature^[^ ^31^ ^]^) necessitates inducing compressive stress of similar magnitude during the GaN growth. Notably, AlN's smaller in‐plane lattice constant (3.112 Å) compared to GaN (3.189 Å) itself entails ≈11 GPa of compressive stress. Hence, at least theoretically, countering the tensile stress is plausible even for direct growth on a relaxed AlN nucleation layer (NL) if just ≈10% of this compressive‐stress source can be retained during growth. Yet, numerous, mostly early, attempts^[^ ^32^, ^33^, ^34^, ^35^ ^]^ to grow buffer‐less large‐area GaN‐on‐Si have culminated in cracks after cooldown, suggesting a failure to control the compressive stress relaxation. Available in situ analysis^[^ ^32^, ^36^ ^]^ showed that without buffers, the stress in GaN was either tensile from the beginning, or if compressive, decayed within the first ≈100 nm, and subsequent growth occurred in the tensile regime. In the last decade, only sporadic reports^[^ ^37^, ^38^, ^39^, ^40^, ^41^, ^42^, ^43^, ^44^ ^]^ of crack‐free buffer‐less GaN‐on‐Si have emerged and these neither provide quantifiable insight into their stress‐structure evolution nor evidence of simultaneous optimization of electronic and thermal properties. Thus, the quantitative understanding needed for reproducible synthesis and optimization of buffer‐less epi‐structures along with the immediate performance advantage for their realization, is unavailable.

## 2 Results and Discussion

### 2.1 Modulation of Stress Relaxation with Growth Parameter

In this work, we designed the individual epilayer thicknesses for buffer‐less GaN/AlN/Si heterostructures based on strain and thermal considerations. It is known that for the AlN nucleation layer, increasing its thickness (*t_AlN_*) reduces its dislocation density.^[^ ^45^ ^]^ However, since the strain state of an AlN NL on Si is constantly tensile during growth, this also increases the strain energy of this layer and, above 200 nm, the potential for cracking during growth.^[^ ^36^ ^]^ Hence, as a trade‐off, t_AlN_ was kept at ≈150 nm in our experiments. In terms of the GaN thickness (*t_GaN_*), simulations can predict the optimum thickness for thermal performance. For GaN HEMTs with a silicon substrate, the aggregate thermal resistance initially decreases with increasing GaN thickness (*t_GaN_*) and then plateaus after ≈500 nm.^[^ ^46^ ^]^ For HEMT membranes (i.e., structures where all layers underneath the GaN are etched away) integrated with synthetic diamond heat‐spreaders, simulations^[^ ^46^ ^]^ suggest little dividend for increasing *t_GaN_* above 1 µm. Large GaN thicknesses are not beneficial from the stress perspective either. Even on graded AlGaN or superlattice buffers the initial compressive stress in the GaN gradually relaxes as the layer thickness increases,^[^ ^17^, ^36^ ^]^ and hence with increasing thickness the cumulative compressive stress will eventually become lower in magnitude than the post‐growth tensile stress. Accounting for all of these factors, we designed *t_GaN_* to be ≈750–800 nm, with the total epi‐thickness of ≤ 1 µm.

**Figure 1:**
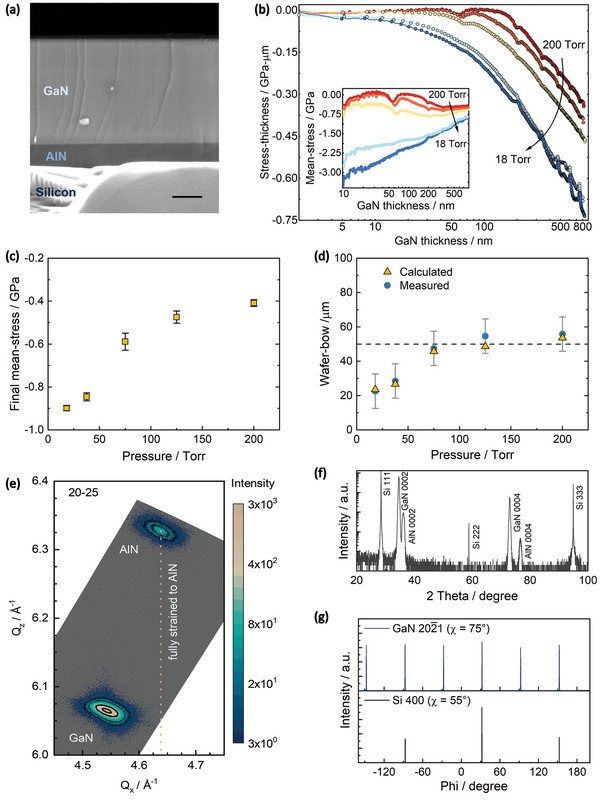
Stress and structural characterisation of GaN/AlN/Si heterostructures. a) Cross‐sectional SEM of a representative structure, scale bar is 200 nm. b) Evolution of stress‐thickness with thickness during the GaN growth at 200, 125, 75, 37.5, and 18 Torr. Inset shows the corresponding mean‐stresses with increasing thickness. Note that the absolute stress‐thicknesses after the AlN growth (i.e. prior to the GaN growth) were similar for all the growths, and in this image, these values have been offset to zero for comparison. c) Mean‐stresses accumulated after 800 nm GaN growth, averaged from multiple runs. d) Post‐growth measured (with a ± 10 µm resolution of the set‐up) and calculated wafer bows at RT. e) XRD RSM around the AlN 20‐25 reflection. f) On‐axis ω‐2θ scan showing peaks from the epilayer and substrate. g) 360° ϕ‐scans for peaks from GaN(20‐21) and Si(400) planes.

For all growths, in situ removal of the native oxide on the six‐inch diameter silicon substrates preceded the growth of the AlN NLs, and both these steps were identical for all samples (see Figure S1, Supporting Information). On these AlN NLs, GaN was directly grown after the change of growth conditions (see Experimental Section). For different samples the reactor pressure (*P_g_*) during GaN growth was varied over an order of magnitude (200, 125, 75, 37.5, and 18 Torr), with all other parameters kept constant. The cross‐sectional secondary electron micrograph (SEM) of an as‐grown structure (**Figure** **1**a) shows the thin GaN epilayer on top of the NL.

During heteroepitaxy, the wafer‐curvature changes due to the stress in the growing layer. Previously, for GaN/step‐graded AlGaN grown on silicon, this real‐time change in wafer‐curvature (which is directly proportional to the layer's stress‐thickness, *σ·t*) can provide valuable insight into stress evolution.^[^ ^17^ ^]^ Using the same formalism, the calculated stress‐thickness versus thickness data for these GaN layers grown at different pressures is shown in Figure 1b. The differential slope of these curves is proportional to the instantaneous stress (*σ_i_*) and the slope from the origin is proportional to the cumulative mean‐stress (*σ_mean_*) at that thickness for the growing GaN layer. The instantaneous stress should be compressive (i.e., *σ·t* versus thickness should be negatively sloped) from the beginning for coherent growth on AlN. The data indicate that at higher pressures, the compressive growth regime does not start from the beginning, but the thickness for its onset is progressively shortened as *P_g_* is reduced. This is further confirmed from the evolution of *σ_mean_* with thickness (inset to Figure 1b). For 18 Torr, the initial *σ_mean_* has the highest magnitude, and it continuously reduces during growth. This indicates that the successive atomic layers grow with increasingly relaxed lattice constant, in effect continuously decreasing the compressive stress averaged across the entire thickness.^[^ ^17^ ^]^ In contrast, for 200 Torr, the layer becomes marginally more compressive in the initial phase (albeit with a much smaller value), followed by a regime of slow relaxation.

Intermediate behaviour is seen for pressures in between 18 Torr and 200 Torr, revealing that the decay of *σ_mean_* is never large enough to become tensile for any structure. Consequently, as shown in Figure 1c, the final mean‐stress of the GaN layers at the growth temperature systematically changes from (−0.41 ± 0.02) GPa to (−0.90 ± 0.01) GPa with the reduction in *P_g_*. Except for the growth at 200 Torr, these mean‐stresses were sufficient to prevent cracking (see Figure S2, Supporting Information) by countering the subsequent tensile stress during cooldown.

Along with avoiding cracking, high‐throughput automated fabrication requires the wafer‐bow to be ≤50 µm. For a multilayer thin film, the wafer‐bow at room‐temperature (RT) is inversely proportional to the wafer‐curvature (κ), which, in turn, depends on each layer's residual stress as $\kappa =\sum \Delta \kappa_{i}=\sum f\left\{\left(\sigma_{i\_growth}+\sigma_{i\_thermal}\right)\times thickness_{i}\right\}$. Accordingly, using the mean growth‐stresses after 800 nm GaN growth ($\sigma_{i\_growth}$) from Figure 1c and considering tensile thermal stress values ($\sigma_{i\_thermal}$) from the literature,^[^ ^31^ ^]^ the RT bows were calculated. As plotted in Figure 1d, predicted values closely agree with the measured data, showing that simply controlling the GaN growth stress enabled the tuning of the post‐growth wafer bow. This confirms that with a wafer bow of (48 ± 10) µm for *P_g_* = 75 Torr, and lower bows for lower *P_g_* values, the majority of the buffer‐less GaN‐on‐Si structures of this series comply with the requirements for batch processing in CMOS fabs. In addition, shedding the buffer layers from the growth sequence provided substantial reduction in energy, material, and runtime (see Figure S3, Supporting Information).

Figure 1e shows an X‐ray diffraction (XRD) reciprocal‐space map (RSM) around the asymmetric 20‐25 AlN reflection for the epi‐structure with the GaN layer grown at 75 Torr. Only reflections corresponding to AlN and GaN are observed, with no evidence observable for any ternary phase. Here, a smaller centroid *Q_x_* of the GaN peak compared to AlN indicates a larger average in‐plane lattice constant, consistent with the gradual relaxation observed during growth. For the same structure, the XRD ω‐2θ scan in Figure 1f features only 000l peaks of the nitrides and 111 and higher order peaks of the silicon substrate, establishing GaN[0001] ∥ Si[111]. Furthermore, only six sharp peaks are seen in the 360° azimuthal scan of the skew‐symmetric reflection of GaN (Figure 1g), confirming a single‐domain rotational alignment of the hexagonal unit cells on the cubic substrate. Altogether, these scans prove that along with stress‐balancing, epitaxially preserving a sixfold atom arrangement of wurtzite nitrides on the threefold symmetric Si(111) surface with the wanted orientation of GaN[11‐20] ∥ Si[‐110] does not require any buffers.

**Figure 2:**
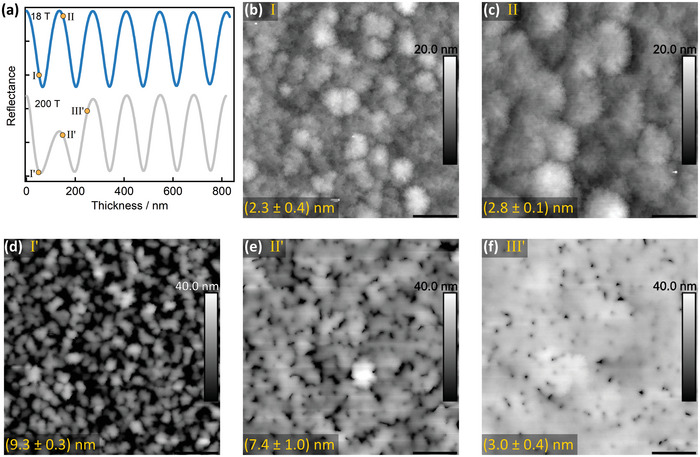
Real‐time reflectance transients and their correlation with surface topography of the GaN layer. a) Oscillations in reflectance transients acquired during 800 nm GaN growths at 18 Torr and 200 Torr, respectively. Note that the data has been offset in intensity (without any multiplication) for clarity. Marked points denote the stage at which additional growth runs were terminated to inspect surface topography. b,c) are 20 × 20 µm AFM images at different stage for growths at 18 Torr, whereas (d–f) are AFM images for GaN grown at 200 Torr. The z‐scale for (b,c) is 20 nm, and for (d–f) is 40 nm. The mean rms roughness (in nm) from three positions on the wafer is annotated for each AFM scan. The scale bar in (b–f) is 4 µm.

We subsequently considered the growth regimes to identify the mechanism behind the difference in strain relaxation among different structures. **Figure** **2**a shows the reflectance data acquired during the growth of the GaN layers for the highest and lowest pressures. Fabry‐Perot oscillations are visible in both, arising from the interference of the beams reflected from the GaN surface and the AlN/Si interface beneath. For *P_g_* = 18 Torr, the peak‐to‐peak magnitudes are the same for all periods. However, for the highest pressure, the initial sharp reduction is followed by gradual recovery (see Figure S4, Supporting Information for the intermediate behaviors at *P_g_* = 37.5 Torr to 125 Torr). To examine the corresponding evolution of topography at different stages, additional growth runs were terminated at the thicknesses marked in Figure 2a. As seen in the atomic force microscopy (AFM) image in Figure 2b, by point I after nominally ≈50 nm of growth at 18 Torr, the GaN layer is already continuous and completely covers the NL. This flat morphology persists as growth progresses (point II, ≈150 nm nominal thickness), along with a marginal increase in the lateral dimensions of the features (Figure 2c). In contrast, isolated islands dominate the morphology at point I' for the growth at 200 Torr (Figure 2d). At this pressure, even at point II', a considerable fraction of the layer is still uncoalesced (represented by dark areas in Figure 2e). The merger of the islands requires a further progression of growth, as seen in Figure 2f. This explains the reflectance evolution seen in Figure 2a as the side facets of the islands would have diffusely scattered the incident laser beam causing a diminished detected intensity in the initial phase. Nonetheless, all the surfaces were smooth at the final layer thickness, with the sub‐nm roughness required for abrupt heterojunctions (see Figure S5, Supporting Information).

**Figure 3:**
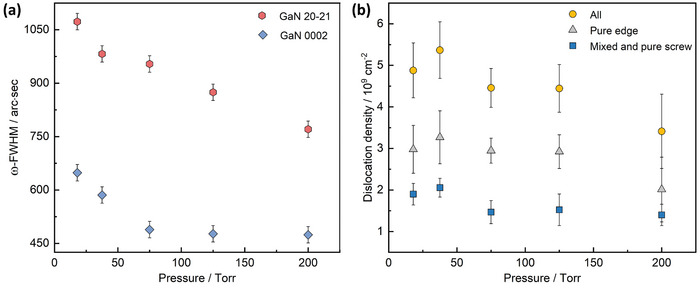
Dependence of structural properties of the GaN layer on growth pressure. a) Variation in FWHM of HRXRD skew‐symmetric 20‐21 and symmetric 0002 ω‐scan peaks of the GaN layer with growth pressure. The error bars represent the intrinsic broadening of the goniometer. b) Variation in the density of the total and individual dislocation density at the surface of the GaN layers grown at different pressures. The error bars represent the standard deviations calculated from multiple positions in the same wafer.

To estimate the threading dislocation density in these [0001] oriented epilayers, full‐width at half maxima (FWHM) of HRXRD ω‐scans for skew‐symmetric 20‐21 and on‐axis 0002 reflections were assessed next. The measured FWHM values in **Figure** **3**a show that as *P_g_* increases, the 20‐21 peak FWHM gradually reduces from 1073 arcsecs to 771 arcsecs. However, after an initial reduction from 648 to 489 arcsecs, the 0002 peak FWHM does not decrease further for pressures higher than 75 Torr. Qualitatively, lower peak broadening indicates smaller in‐plane twist and better out‐of‐plane tilt alignment among the individual crystallites, respectively. Quantitatively, from the 20‐21 peak FWHM, the density of dislocations with edge‐character (pure‐edge and mixed), which are the most prevalent in wurtzite nitrides (usually < 2% are pure‐screw types), can be estimated^[^ ^47^ ^]^ as $D_{e}=\frac{{\left(FWHM\right)}^{2}}{4.35b_{edge}^{2}\;}$ where *b* is the Burgers vector. This suggests an anticorrelated change in the edge‐type dislocation density from 6 × 10^9^ cm^−2^ to 3 × 10^9^ cm^−2^ with the investigated growth pressures. Note that the XRD signal is collected from the entire GaN layer, and the calculated values represent a weighted average across the thickness. Among these, dislocations threading to the surface will directly affect the functionality of the subsequently grown HEMT channel layer. To quantify the TD density at the surface of these templates and distinguish different dislocation types, pits created by the surface terminations of dislocations were imaged by AFM^[^ ^47^ ^]^(see Figure S6, Supporting Information). The scan results are summarized in Figure 3b. A general trend of a reduction in the total dislocation density from (4.9 ± 0.7) × 10^9^ cm^−2^ to (3.4 ± 0.9) × 10^9^ cm^−2^ with increase in *P_g_* can be confirmed with pure edge dislocations accounting for 59–66% of the total densities. It must be emphasized that these dislocation densities are very similar to those achieved for GaN grown with different thick buffers.^[^ ^17^, ^21^, ^22^ ^]^

These observations open up multiple avenues to control the involved stress evolutions. The 2.5% lattice mismatch between GaN and AlN provides a driving force for GaN to nucleate and grow as discrete islands. From Figure 2a, it is evident that the duration of this island‐mode growth increases with increasing reactor pressure. Also, little change in stress‐thickness before island coalescence (in Figure 1b) suggests that the tensile stress associated with coalescence itself (for the observed domain sizes, a maximum theoretical value on the order of GPa^[^ ^48^ ^]^ can be estimated for GaN's modulus) largely compensates the compressive stress arising from the lattice‐mismatched growth during this period. Post‐coalescence, the observed gradual decay of compressive stress is characteristically similar to that often associated with layer‐by‐layer growth of GaN on AlGaN and originates from dislocation climb driven by compressive stress.^[^ ^36^ ^]^ While dislocation climb facilitates annihilation of dislocations with opposite Burgers vectors, for our structures, this mechanism is active only during the post‐coalescence compressively‐strained phase. Hence, it cannot be responsible for the reduction in dislocation density at higher growth pressures with increasingly delayed coalescence. Deliberate islanding has been key to achieving low dislocation density in GaN directly grown on sapphire. This acts by bending dislocations toward the facets which minimize the systems’ free energy.^[^ ^49^ ^]^ It is likely that a similar mechanism is involved in our samples up until coalescence, instead of climb. This appears to be the mechanism for reducing dislocations albeit at the expense of accumulating less compressive stress during GaN growth. A transmission electron microscopy study is planned to confirm this and will be published separately.

Importantly, while the reactor pressure was implemented herein to modulate the coalescence, we note that recently both Lee et al.^[^ ^43^ ^]^ and Zhan et al.^[^ ^44^ ^]^ have successfully grown crack‐free buffer‐less GaN‐on‐Si with AlN NL by using a very large V/III ratio (≥15 000) during the initial growth phase. In fact, parameters like the V/III ratio, pressure, temperature, and growth rate are known control parameters used during the 3D‐2D transformation of low‐defect GaN‐on‐sapphire growth. In the future, systematic exploration of all these correlated parameters during GaN on AlN NL growth can be expected to further expand the growth window for buffer‐less nitrides on silicon.

### 2.2 Analysis of Thermal Resistance for Buffer‐Less GaN‐on‐Si

**Figure 4:**
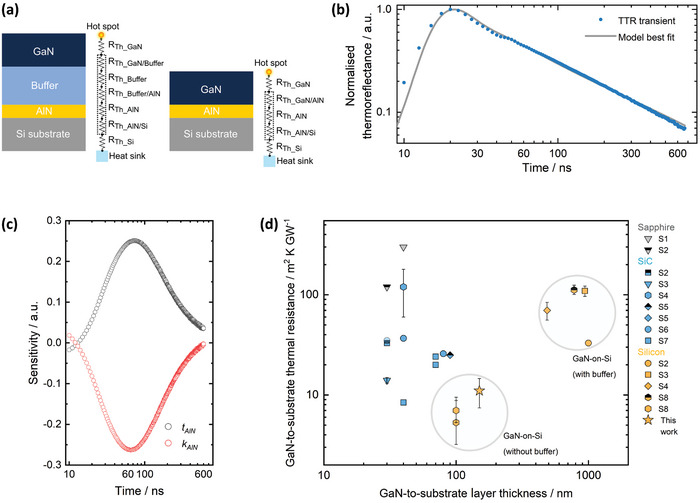
Thermal characterisation of GaN/AlN/Si heterostructures. a) Schematic of the thermal resistances in the heat diffusion path from the channel hotspot to the heat‐sink under the silicon. The individual resistances between the GaN and the Si substrate are lumped, which shows our buffer‐less design possesses fewer thermal elements than the conventional one. The interface resistances will likely be dominated by disorder rather than the phonon density mismatch in both. b) Representative measured TTR trace for the sample for which the GaN layer was grown at 75 Torr, normalised to the peak thermoreflectance. Modelled best fit (solid line) closely approximates the experimental data. c) Sensitivity analysis with respect to the variation in k_AlN_ and t_AlN_. d) Comparing literature reports across heteroepitaxial GaN on sapphire, SiC, and Si, the measured sample has one of the lowest thermal resistances between the GaN layer and the non‐native substrate (references for the data are listed in Table S1, Supporting Information).

For our sample series, we suggest that the GaN layer grown at 75 Torr presents the best balance of stress‐management and micro‐structural properties and experiments henceforth refer to GaN grown at this pressure. To evaluate the thermal transport across this GaN/AlN/Si heterostructure we used the nanosecond transient thermoreflectance (TTR) technique.^[^ ^27^, ^50^, ^51^ ^]^ This non‐invasive method involves a nanosecond pulsed laser to heat the sample surface and a continuous probe laser to measure the subsequent evolution of sample temperature with constant probing of surface Fresnel reflection (see Experimental Section). In multi‐layered structures, this evolution depends on the thermal diffusivity of each layer through which the heat diffuses and possible thermal boundary resistances (TBR) at the interfaces between different materials. For our structure (**Figure** **4**a), the thermal resistances associated with the AlN layer are the only unknown parameters in the cumulative thermal resistance between GaN and substrate. We treat these as a lumped thermal boundary resistance (*TBR_eff_*),^[^ ^18^ ^]^ summing the AlN layer thermal resistance and the GaN/AlN and AlN/Si interface TBRs, $TBR_{eff}=\frac{t_{AlN}}{k_{AlN}}+TBR_{GaN/AlN}+TBR_{AlN/Si}$, where *t_AlN_* and *k*_AlN_ are AlN layer thickness and thermal conductivity, respectively. For thin layers, it is impossible to accurately separate the intrinsic layer thermal resistance from the TBR contribution, so instead, we use an effective value for the AlN layer (*k_AlN_eff_*), which includes the TBR contribution, i.e., $TBR_{eff}=\frac{t_{AlN}}{k_{AlN\_eff}}$.

TTR transients were acquired from four different positions on the sample, and a representative normalized curve is shown in Figure 4b. The experimental data were least‐squared fitted with an analytical transmission‐line axis‐symmetric heat diffusion model^[^ ^52^ ^]^ with $k_{AlN\_eff}$ as the fitted variable (see Experimental Section). Figure 4b shows the simulated best fit for the representative transient. The sensitivity (*S_x_*) of the thermoreflectance (*R*) to the fitting parameters (*x*) was also calculated and this is plotted in Figure 4c where $S_{x}=\frac{\partial \{\ln(R)\}}{\partial \{\ln(x)\}}$. A high sensitivity to both $k_{AlN\_eff}$ and *t_AlN_* can be seen in the ≈20–300 ns period. However, as the uncertainty in calibrated *t_AlN_* is minimal, this gives confidence in the extracted $k_{AlN\_eff}$. Based on the 150 nm thick AlN, we calculated the (mean ± std. dev.) *TBR_eff_* for this sample to be (11 ± 4) m^2^ K^−1^ GW^−1^. Theoretically, the sum of the GaN/AlN^[^ ^53^ ^]^ and AlN/Si^[^ ^26^ ^]^ interfacial resistances (predicted by the diffuse mismatch model) and the AlN layer thermal resistance (considering its bulk conductivity^[^ ^26^ ^]^) is predicted to be ≈2 m^2^ K^−1^ GW^−1^. However, in practice, the AlN's large dislocation density and limited thickness (compared to phonons with longer mean free path) will significantly lower its thermal conductivity compared to bulk material. Altogether, it is likely the best experimentally achievable *TBR_eff_* will be higher than this theoretical value. This is evident from Figure 4d where we plot cumulative thermal resistance between the GaN layer and underlying non‐native substrate against layer thickness given in the literature, as a benchmarking exercise. Clearly, compared to most GaN‐on‐Si grown with conventional buffers, our structure possesses almost an order‐of‐magnitude smaller thermal resistance between the GaN layer and the silicon. In fact, the measured *TBR_eff_* is among the lowest values for heteroepitaxial GaN across all substrates. This improvement at the material level is expected to substantially benefit heat dissipation from fabricated devices., E.g., thermal simulation of standard multi‐finger HEMTs based on our buffer‐less heterostructure shows tens of degrees Celsius lower temperature in the GaN layer in comparison to the conventional GaN‐on‐Si design for identical power dissipation in channel and base temperature of substrate (see Figure S7, Supporting Information).

To date, efforts to improve the heat extraction of GaN‐on‐Si HEMTs have focused primarily on two post‐epitaxy pathways. One approach aims to bring the heat‐extraction boundary closer to the NL, either by embedding microfluidics^[^ ^54^ ^]^ in silicon with active cooling or micromachining the silicon substrate with metal‐filled trench vias.^[^ ^55^ ^]^ However, the buffer and its large thermal resistance still remain in both cases. Alternatively, all layers beneath the GaN are removed by etching, and then, high thermal conductivity diamond is deposited on the backside as a heat spreader.^[^ ^50^, ^56^ ^]^ However, along with unnecessary growth and then removal of nitride epilayers, the removal of buffer layers may cause problems with stress management. If required, buffer‐less GaN‐on‐Si would be amenable to all these post‐growth approaches to reduce its channel‐to‐heat‐sink thermal resistance further, but without the current limitations. Local silicon substrate removal should also eliminate RF coupling with silicon and substantially enhance the breakdown voltage for the same epilayer thickness.^[^ ^57^ ^]^

### 2.3 Room‐Temperature Electronic Properties and Low‐Temperature Magneto‐Transport of AlGaN/GaN Heterostructures

**Figure 5:**
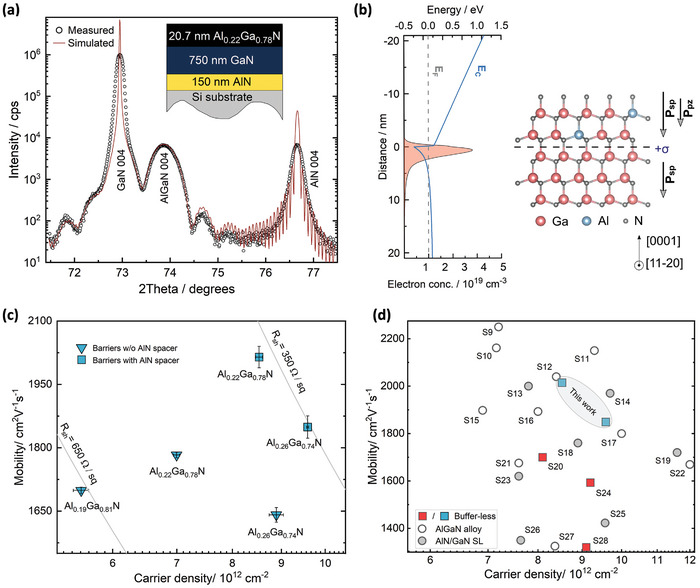
Interfacial confinement and room‐temperature (RT) transport properties of the 2DEG. a) Close agreement between measured XRD ω‐2θ data of the symmetric (004) reflection with simulation confirms the growth and abruptness of the heterojunction (the simulated structure is shown in the inset). b) Simulated conduction band edge of the as‐grown type‐I structure shows that the distribution of electrons is primarily confined on the GaN side of the interface. A schematic of the Ga‐polar lattice is shown with the direction of the polarisation fields. c) Hall‐effect measured variation in 2DEG electron density and mobility with barrier composition for designs without and with the AlN spacer. The error bars represent standard error the mean. d) Benchmark plot showing the comparison with previously reported Hall‐effect values for GaN‐on‐Si epistructures with buffer‐less and various buffer schemes (references for the data are listed in Table S2, Supporting Information) confirms that the 2DEGs studied in this work are among the best with simultaneous high mobility and carrier density.

Next, to create heterojunctions on our buffer‐less GaN‐on‐Si templates, in a separate run, a ≈20 nm Al_0.22_Ga_0.78_N layer was grown after the optimized GaN layer. The 0004 ω‐2θ XRD data of this structure in **Figure** **5**a show well‐defined satellite peaks around GaN and AlGaN peaks, corroborating a sharp interface between the two top layers. XRD RSM confirmed that the Al_0.22_Ga_0.78_N layer is pseudomorphically strained to the underlying GaN (see Figure S8, Supporting Information).

This biaxial strain state in Al_0.22_Ga_0.78_N should generate a piezoelectric polarization (***P_pz_***) which is further supplemented by the difference in spontaneous polarization (***P_sp_***) between Al_0.22_Ga_0.78_N and GaN. As shown in the simulated energy band diagram (Figure 5b), this polarization sheet charge at the interface^[^ ^4^ ^]^ along with the heterojunction's large conduction band offset should confine electrons as a 2DEG in GaN. This was verified by low‐field Hall‐effect measurements of this heterostructure, at room temperature (RT) which showed an average charge carrier density (*n_s_*) of 7.0 × 10^12^ cm^−^^2^ and mobility (*µ*) of 1783 cm^2^ V^−1^ s^−1^ along with a negative Hall coefficient. Moreover, an almost constant carrier density along with a continuously increasing mobility with the reduction in temperature (see Figure S9, Supporting Information), confirmed the well‐known signatures of a 2DEG.

For 2DEGs, mobility remains the key evaluator of electrical cleanliness^[^ ^58^ ^]^ and requires careful optimization. To test the variation of *µ* with *n_s_* for our structures, we grew additional samples with barrier compositions of Al_0.19_Ga_0.81_N and Al_0.26_Ga_0.74_N. Among these three samples (triangular symbols in Figure 5c), *n_s_* showed a progressive increase from 5.6 × 10^12^ cm^−^^2^ to 8.9 × 10^12^ cm^−^^2^ with increasing AlN mole fraction (x), consistent with increased spontaneous and piezoelectric polarization contributions.^[^ ^4^ ^]^ The fact that we observe a lower electron mobility for *x* = 0.26 compared to *x* = 0.22 suggests that scatterers which are more screened^[^ ^59^, ^60^ ^]^ at higher carrier densities such as charged dislocations or background ionized impurities, are not dominant for *x* ≥ 0.22 barriers on our template. Instead, in this carrier density regime, increased interface roughness and alloy scattering is likely the cause for mobility reduction as the wavefunction penetrates deeper into the barrier.^[^ ^61^ ^]^ To alleviate this penetration, we introduced a ≈1 nm AlN spacer between the channel and the AlGaN barrier. For samples with such a spacer and with barrier compositions of Al_0.22_Ga_0.78_N and Al_0.26_Ga_0.74_N we observed a significant increase in mobility to 2015 and 1849 cm^2^ V^−1^ s^−1^ (square symbols in Figure 5c), respectively. Simultaneously, *n_s_* increased to 8.5 × 10^12^ cm^−^^2^ and 9.6 × 10^12^ cm^−^^2^ due to a polarization boost from the spacer. A comparison of these samples with other reported AlGaN/GaN HEMT heterostructures on silicon is shown in Figure 5d. The overview shows that our RT Hall‐effect mobilities are significantly higher than those previously achieved without buffers. Also, along with larger or comparable electron densities, our mobility values are among the highest reported for GaN‐on‐Si, including various buffer schemes on silicon that amass up to 8 µm of total epi‐thickness (see Table S2, Supporting Information). The data in Figure 5d provide clear evidence that neither buffers nor thick epilayers are needed to realize high‐quality nitride heterostructures with high 2DEG mobility and density. Furthermore, the different carrier densities in Figure 5c demonstrate that the standard technique of altering the 2DEG carrier density by tuning the barrier composition for buffered structures is equally applicable to our buffer‐less design. Hence depending on requirements, carrier densities lower or higher than those reported in this study can be achieved by simply decreasing or increasing “x” of the Al_x_Ga_1−x_N barrier. Notably, the buffer‐less design has no impact on the electrical nature of the GaN layer. For example, the resistance of the GaN layer without any AlGaN barrier on top was too high to be measurable using Hall‐effect measurements. Moreover, capacitance‐voltage measurements showed that all the carriers are located at the barrier/channel interface in the heterostructures with barriers, and the background carrier concentration in the GaN layer is ≈10^15^ cm^−3^ (see Figure S10, Supporting Information). This confirms its high‐resistivity nature, even without deep‐level doping.

**Figure 6:**
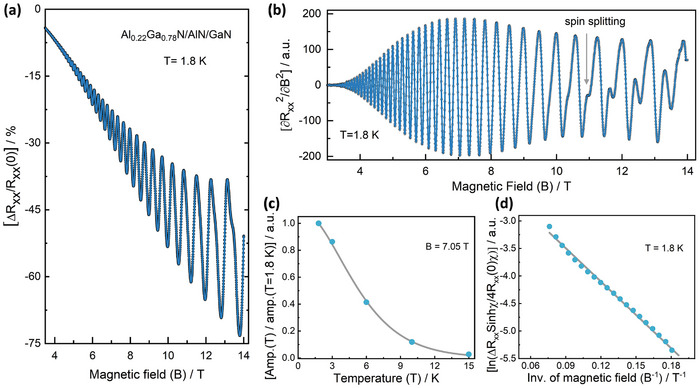
Quantum Hall signatures in the AlGaN/AlN/GaN 2DEG at low temperatures. a) Change in longitudinal magnetoresistance (ΔRxx) with increasing magnetic field, B, at 1.8 K. The data has been normalised with respect to the value at B = 0 Ti.e. R_xx_(0). Distinct Shubnikov de Haas (SdH) oscillations can be observed along with a parabolic background. b) The second derivative of the raw magnetoresistance data plotted in (a) removes the background and shows only the changing SdH amplitudes with magnetic fields. After an initial increase, the peak‐to‐peak amplitudes starts to decrease from B ≈7.5 T. Moreover, from ≈10 T, a secondary oscillation is seen whose amplitude increases with B. c) Temperature dependence of the normalised magnetoresistance peak (with respect to the value at 1.8 K) at constant magnetic field were used to extract the effective carrier mass. A representative fit (solid line) for the data (solid circles) at 7.05 T is shown. d) Representative Dingle plot for the oscillation amplitude peaks from ≈5.5 T to ≈13 T and considering m* = 0.210 m_0_ (obtained from 6c). The straight line is a single‐parameter fit to the data (solid circles) to extract the associated quantum scattering time from the slope.

Low‐temperature quantum magneto‐transport is a more challenging metric of high‐quality for 2DEGs in modulation doped^[^ ^58^ ^]^ or undoped polar^[^ ^62^ ^]^ heterostructures., E.g., if scattering is sufficiently low (i.e., *ω_c_τ* > 1, where *ω_c_* is the cyclotron frequency and *τ* is the carrier relaxation time), the 2DEG's longitudinal resistance (*R_xx_*) becomes oscillatory in increasing magnetic field (i.e Shubnikov‐de‐Haas (SdH) oscillations are observed). The perception that delicate magneto transport requires high‐quality epilayers with low defect density has compelled the nitride community to restrict such measurements primarily on samples grown on bulk GaN,^[^ ^63^ ^]^ SiC,^[^ ^64^ ^]^ or sapphire.^[^ ^65^ ^]^ The high mobilities in Figure 5 indicated that such features could be potentially observable in our structures. Hence, we investigated magneto‐transport in the Al_0.22_Ga_0.78_N/AlN/GaN structure with the highest mobility. As shown in the low‐temperature normalized magnetoresistance (MR) trace of **Figure** **6**a, distinct SdH oscillations with progressively increasing amplitudes with the magnetic field (*B*) were seen. These oscillations appeared superimposed on a negative MR with parabolic dependence on *B*. Collectively, this resulted in a substantial change in magnetoresistance at larger fields, reaching ≈75% by 14 T. Figure 6b shows the data after removing the *B^2^*‐dependent background by double differentiation of the raw MR data.^[^ ^66^ ^]^ In addition to the main SdH oscillations which begin at ≈4 T, a secondary oscillation is prominent in the spectra above 10 T. The respective periodicities of the two oscillations above 10 T with *B^−1^* yields slightly distinct sheet carrier densities behind their origins (see Figure S11, Supporting Information). The calculated total *n* was ≈8.8 × 10^12^ cm^−^^2^, which is consistent with the low‐field Hall effect data for this wafer (8.5 × 10^12^ cm^−^^2^). This agreement validates the strong 2D confinement of the channel carriers. Moreover, this proves that the spin degeneracy of the single subband in the QW was removed, yielding two spin‐split subbands^[^ ^64^, ^65^ ^]^ (*n*↑ and *n*↓). Its origin is likely the Rashba spin‐orbit coupling effect^[^ ^67^ ^]^ in this noncentrosymmetric system, enhanced by the built‐in interfacial electric field perpendicular to the QW.

For these measurements, increasing the temperatures from 1.8 to 15 K showed a gradual damping of the SdH oscillations. This temperature dependence of the peak amplitudes was used^[^ ^68^ ^]^ to extract the electron's effective mass (m^*^) in the subband. For these fits, the peaks between 6 and 8 T were selected as the oscillations in Figure 6b were the strongest in this regime. A representative fit (for 7.05 T) is shown in Figure 6c. The estimated m^*^ values were between (0.208 ± 0.008)m_0_ and (0.215 ± 0.005)m_0_, consistent with those obtained from different techniques in the literature.^[^ ^69^ ^]^ Also, based on these effective masses, the quantum scattering time (*τ_q_*) was extracted from the slope of the Dingle plots (Figure 6d). *τ_q_* is a measure of Landau level broadening and is affected by scattering events over all angles (unlike the large‐angle scattering dominated transport lifetime that governs the Hall‐effect mobility. Values between 0.184 and 0.191 ps were obtained for *τ_q_*, a relatively large lifetime compared to previous reports.^[^ ^70^ ^]^ Effectively, this yields a quantum mobility, µ_q_ (=*e*τ_*q*_/m* ), of ≈1550 cm^2^ V^−1^ s^−1^.

These observations have important implications for III‐nitrides. Compared to the widely investigated homoepitaxial III‐arsenides and phosphides, QWs in GaN heterojunctions present a g^*^‐factor close to that of free electron, an m^*^ three times heavier than GaAs but comparable Rashba spin‐orbit coupling. Buffer‐less GaN‐on‐Si now presents an industrially scalable, technologically relevant platform for probing and exploiting a unique regime of mesoscopic physics.

## 3 Conclusion and Outlook

In summary, we quantitatively established that by controlling the coalescence of the GaN layer, the lattice and thermal mismatch challenges of III‐nitride heteroepitaxy on Si can be overcome even without buffer layers. Benefiting from the absence of thermally poor conductive buffers, our structures provide an ultra‐high thermally conductive pathway for heat removal. The excellent crystal quality of these buffer‐less epilayers is further evidenced by the simultaneous realization of state‐of‐the‐art 2DEG mobility and large sheet carrier density with AlGaN/GaN heterojunctions.

It is worth mentioning that thickness variations of our buffer‐less design can enhance application‐specific functionalities., E.g., AlN provides superior channel confinement compared to AlGaN back‐barriers. Hence, as a possibility we suggest that a reduction in GaN thickness could enhance this effect further, providing the short‐channel immunity needed for ultra‐scaled RF transistors and ultimately paving the realization of an AlN/GaN/AlN quantum well field‐effect transistor^[^ ^71^ ^]^ on Si. On the other hand, for power devices, it may be possible to thicken the GaN drift layer further to increase the breakdown voltage by screening the fields from the substrate. Altogether, a new regime of exploration and implementation of lower‐cost, sustainably‐produced GaN‐based heterostructures for high‐performance HEMTs and novel quantum states in the 2D limit is envisaged.

## 4 Experimental Section

### Epitaxial Growth and In Situ Characterization

The epitaxial structures were grown in an industrial‐grade rotating‐disc (≈1000 rpm) MOVPE reactor (Veeco Propel) with trimethylgallium (7N pure, source: Pegasus chemicals), trimethylaluminum (7N pure, source: Dow chemicals), and ammonia (6N5 pure, source: BOC) as the precursors and hydrogen (source: BOC, purified to < 50 ppb through a purifier (make: Entegris)) as the carrier gas. 1 mm thick, epi‐ready p‐type six‐inch (i.e., 150 mm) diameter Si(111) wafers (source: Shin‐Etsu Handotai) were directly loaded into the reactor without any chemical treatment. After in situ removal of the native oxide by heating under hydrogen, the AlN NLs were grown in two temperature steps, both at pressure of 75 Torr. The first stage was grown at 750 °C temperature, and after a fast temperature ramp with growth continuing, the second stage (≈140 nm) was grown at 1070 °C. Before GaN growth, the temperature was ramped down to 1050 °C and the reactor pressure was changed to the value intended for that sample, and these parameters were kept fixed during the entire GaN growth duration. The V/III molar ratio was kept low at ≈75 during AlN growth and ≈1500 during GaN growth. The temperature, reflectance, and curvature of the wafers were continuously measured in real‐time during growth with the integrated Veeco DRT‐210 process monitoring tool, which uses a narrow‐bandwidth 650 nm laser. All the temperatures mentioned were emissivity‐corrected wafer temperatures.

### Structural and Topographical Characterization

A ZEISS scanning electron microscope with a field emission gun (GeminiSEM 300) operated at 2 kV with 200 nA beam current was used to acquire the cross‐sectional SE image of the structure. The crystalline quality of the epilayers and the epilayer‐substrate alignments were measured in symmetric and skew‐symmetric geometry in a four‐circle horizontal high‐resolution X‐ray diffractometer (Philips PW3050/65 X'Pert Pro) with monochromated CuKα_1_ radiation and a proportional counter. The thickness of the thin AlGaN barrier layers was determined by comparing the measured ω‐2θ curves with simulations obtained from the PANalytical Epitaxy software. The RSM of asymmetric XRD reflections of the epilayers were collected in a four‐circle vertical high‐resolution diffractometer (Panalytical Empyrean) with monochromated CuKα_1_ radiation and a position‐sensitive detector (PIXcel^3D^). The sample topographies were assessed in a Bruker Dimension Icon Pro AFM in Peakforce Tapping mode using commercial SCANASYST‐AIR tips (nominally having 2 nm tip radius and 0.4 Nm^−1^ spring constant). The acquired raw data was processed with the Bruker NanoScope Analysis v1.9 software.

### Thermal Characterization

All the TTR measurements were taken at room temperature. A 10 ns, 355 nm frequency tripled Nd:YAG pump laser with a 30 kHz repetition rate was defocused on the sample surface (≈40 µm FWHM Gaussian profile) to induce heating, and a focused CW 320 nm laser was used to monitor the reflectance. Using above‐bandgap UV lasers (absorption depth < 100 nm for GaN) ensured that the heating and probing were restricted to the GaN surface. For fitting the TTR data with the model for thermal transport, thickness (*t*), material density (*ρ*), specific heat capacity (*C*), and thermal conductivity (*k*) of each layer were treated as fixed parameters. Values^[^ ^27^ ^]^ at 298 K were used for material‐dependent parameters, listed below (**Table** **1**).

**Table 1:** Thermal conductivity (*k*), specific heat capacity (*C*), and material density (*ρ*) for GaN, AlN, and Si. “*t*” denotes the thickness of each layer used in the investigated samples.

Layer	*k* / W m^−1^ K^−1^	*C* / J Kg^−1^ K^−1^	*ρ* / Kg m^−3^	*t* / nm
GaN	140	415	6150	750
AlN	*Fitted*	730	3260	150
Si	149	700	2330	10^6^

### Energy Band Calculations

The energy band diagram of the Al_0.2_Ga_0.8_N/GaN heterostructure was calculated by self‐consistent solution of the Schrodinger equation and Poisson equation using the software BandEng (available from https://my.ece.ucsb.edu/mgrundmann/bandeng.htm). The in‐built material properties within the software were used for the simulation.

### Electrical Characterization

The sheet resistance, carrier concentration, and mobility at room temperature and low temperatures (down to 77 K) of all samples were measured in an Ecopia HMS 5000 system with a fixed magnetic field of ± 0.55 T while sourcing 50–150 µA DC current. For these measurements, square 1 cm^2^ samples were processed by conventional photolithography with annealed Ti/Al/Ti/Au ohmic contacts at the corners to conform to the square van der Pauw pattern. The average values from three tested samples of each wafer are reported in the main text. The cryogenic (down to 1.8 K) magneto‐transport measurements were conducted in a Dynacool Physical Property Measurement System (PPMS) by Quantum Design Ltd. with a superconducting magnet of variable field strength (up to 14 T). Magnetoresistances were measured in a four‐probe configuration with 10 µA AC current excitation using standard lock‐in techniques. For all electrical characterizations under magnetic fields, the applied field was perpendicular to the heterostructure.

## Conflict of Interest

The authors declare no conflict of interest.

## Author Contributions

S.G. and R.A.O. conceptualized the investigation. S.G. and A.M.H. did the epitaxial growths and relevant data analysis. S.G., M.F., D.J.W., and R.A.O. conducted the structural and topographical characterizations of the samples and analyzed the collected data. J.W.P., D.F., and M.K. performed the thermal characterization. S.G. carried out the Hall‐effect and low‐temperature magneto‐transport measurements and analyzed the collected data. S.G., M.F., and R.A.O. co‐wrote the manuscript and all the authors commented on it.

## Supporting information



Supporting Information

## Data Availability

The data that support the findings of this study are openly available in the University of Cambridge repository, doi.org/10.17863/CAM.114906. The data from other articles used for the benchmark plots are tabulated in the tables of the Supporting Information.
