# Supplementary material for: Buffer‐Less Gallium Nitride High Electron Mobility Heterostructures on Silicon
Source: Adv Mater. 2025 Jan 23;37(9):2413127. doi: 10.1002/adma.202413127 (PMC11881668; doi:10.1002/adma.202413127)
Supplement: Supplementary file 1 — Supporting Information [file ADMA-37-2413127-s001.docx]

Supporting Information

**Buffer-less Gallium Nitride High Electron Mobility Heterostructures on Silicon**

*Saptarsi Ghosh^1,2,*^, Martin Frentrup^1^, Alexander M. Hinz^1^, James W. Pomeroy^3^, Daniel Field^3^, David J. Wallis^1,4^, Martin Kuball^3^, and Rachel A. Oliver^1^*

^1^Department of Materials Science and Metallurgy, University of Cambridge, Cambridge CB3 0FS, United Kingdom

^2^Department of Electronic and Electrical Engineering, Swansea University, Swansea SA1 8EN, United Kingdom

^3^H.H. Wills Physics Laboratory, University of Bristol, Bristol BS8 1TL, United Kingdom

^4^Centre for High Frequency Engineering, Cardiff University, Cardiff CF24 3AA, United Kingdom

*E-mail: saptarsi.ghosh@swansea.ac.uk

Figure S1(a) shows the evolution of the stress-thickness with increasing thickness during the second stage (≈ 140 nm) of the AlN layer growth for one of the GaN/AlN/Si hetero-structures. The data points (solid symbols) could be fitted with a straight line having a positive slope, which suggests that the stress was constant and tensile. For the five GaN growths shown in Fig. 1a of the main manuscript, the mean stress during the underlying AlN NL growth was (1.46 ± 0.04) GPa.

**Supplementary Figure S1**. (a) Representative stress-thickness curve and (b) topography of the AlN nucleation layers (NL). The scale bar in the image is 1 µm.


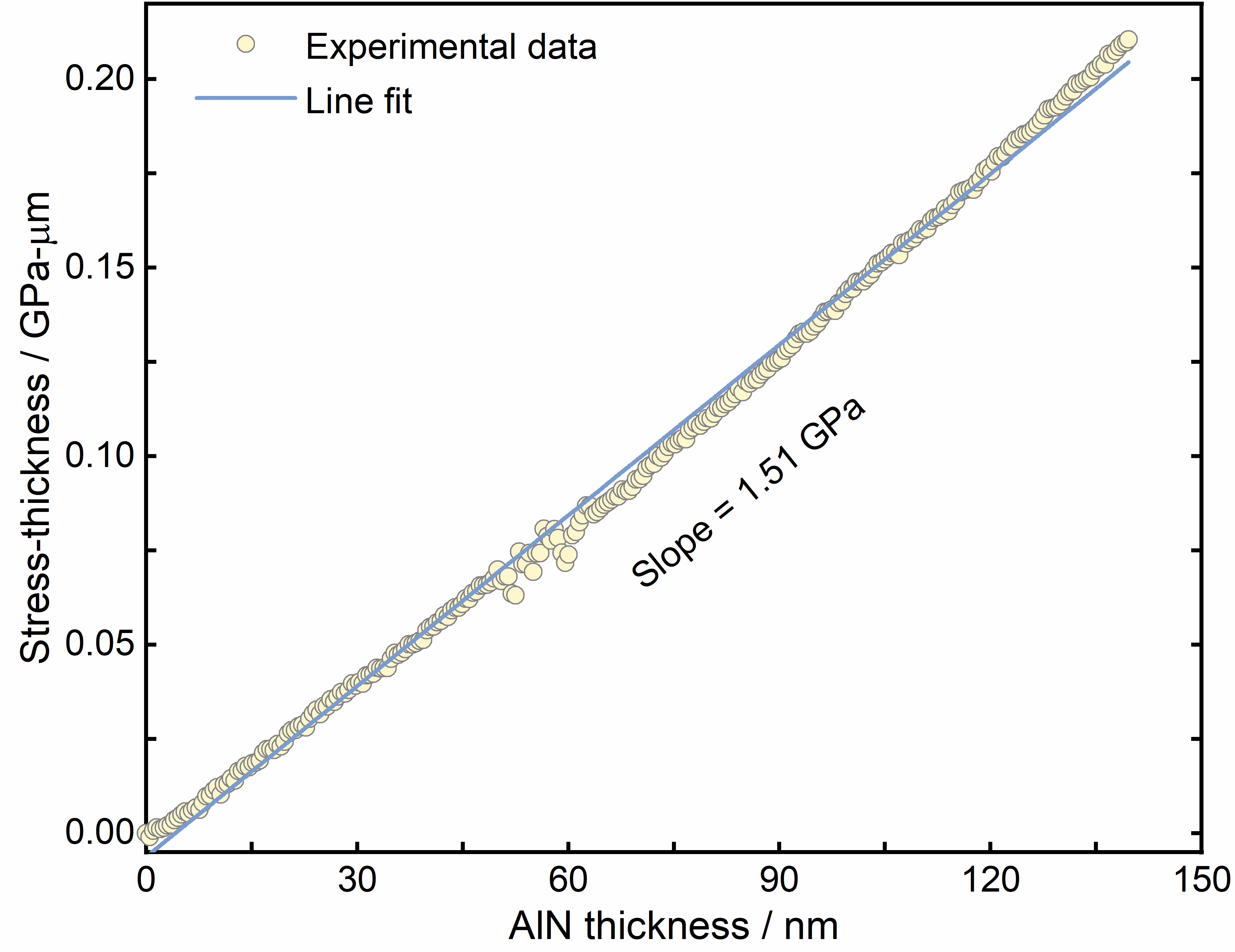


**a**

**b**

0.32 ± 0.03


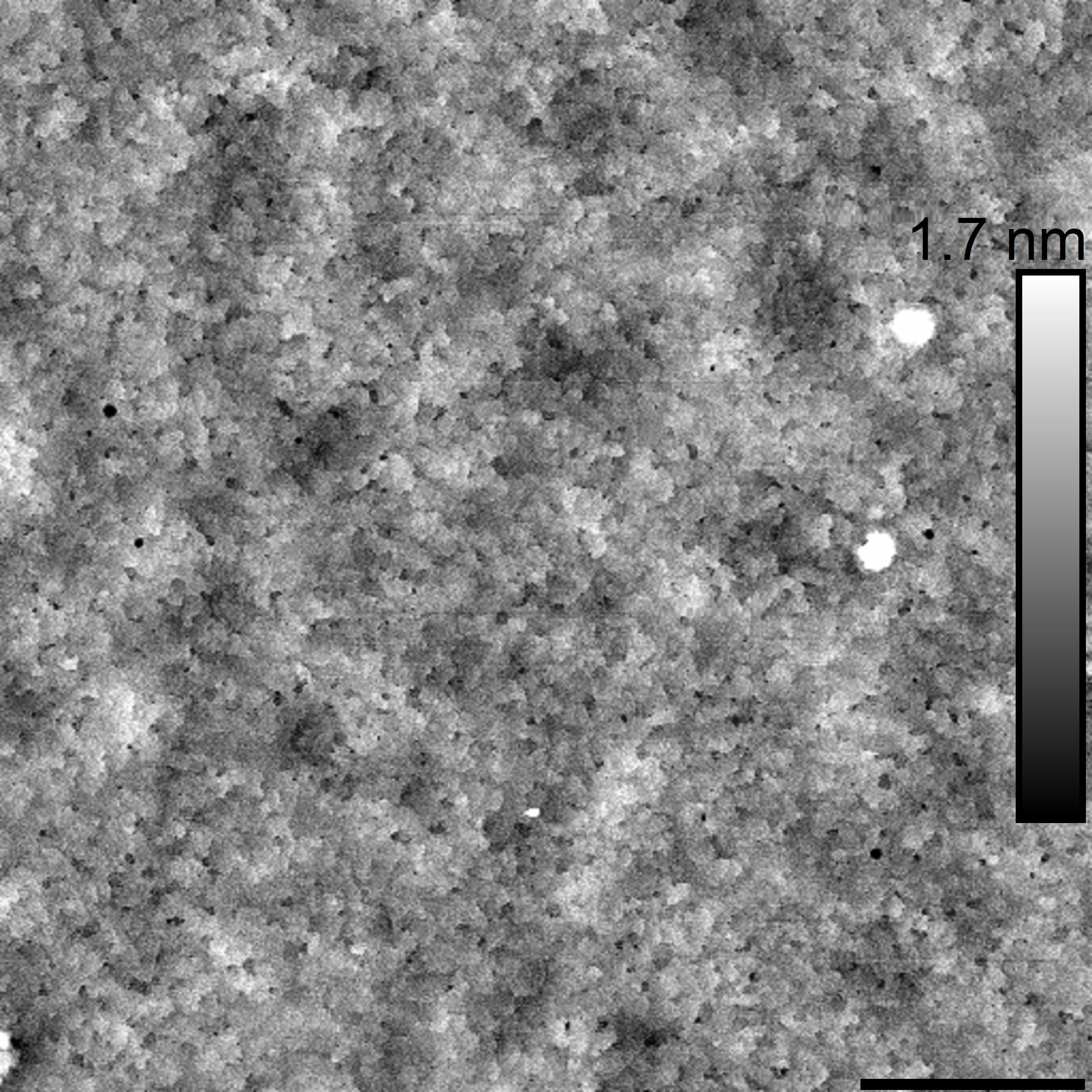


Figure S1(b) shows the surface topography of a representative 5 µm × 5 µm area of an identically grown AlN NL on Si. AFM scans from different regions of the wafer showed sub-nm rms roughness with a mean value of (0.32 ± 0.03) nm.


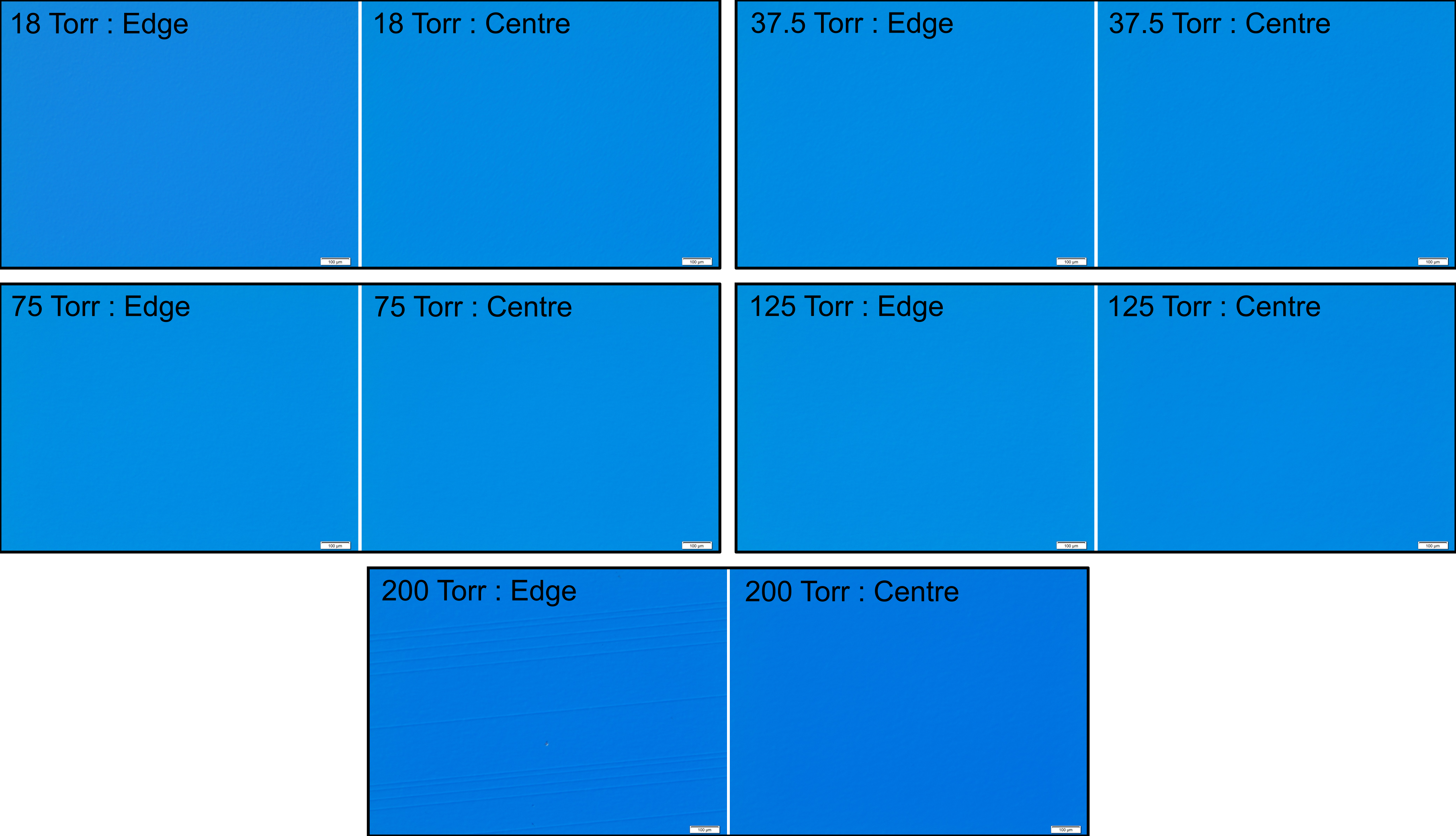


**Supplementary Figure S2**. Larg**e**-area Differential Interface Contrast (DIC) optical micrographs of the surfaces after ≈ 800 nm of GaN growth at different reactor pressures.

Figure S2 shows large-area Differential Interface Contrast (DIC) optical micrographs of the surfaces after ≈ 800 nm of GaN growth at different reactor pressures. Representative images were acquired near the wafers' edge (≈ 1 cm inside) and at the centre. All the surfaces show pit-free mirror-smooth morphology, indicating complete coalescence of these submicron heteroepitaxial layers. The first appearance of cracks can be observed near the edge of the wafer for which the GaN layer was grown at 200 Torr reactor pressure, though they do not propagate to the wafer centre. For growth pressures below this, all the wafers are crack-free. The scale bars in all the images are 100 μm.

**Supplementary Figure S3**. Evolution of reflectance during a representative growth run of 800 nm GaN/ 150 nm AlN on a silicon(111) wafer. The growth steps of in-situ oxide desorption, AlN growth, GaN growth, and post-growth cooldown are indicated.


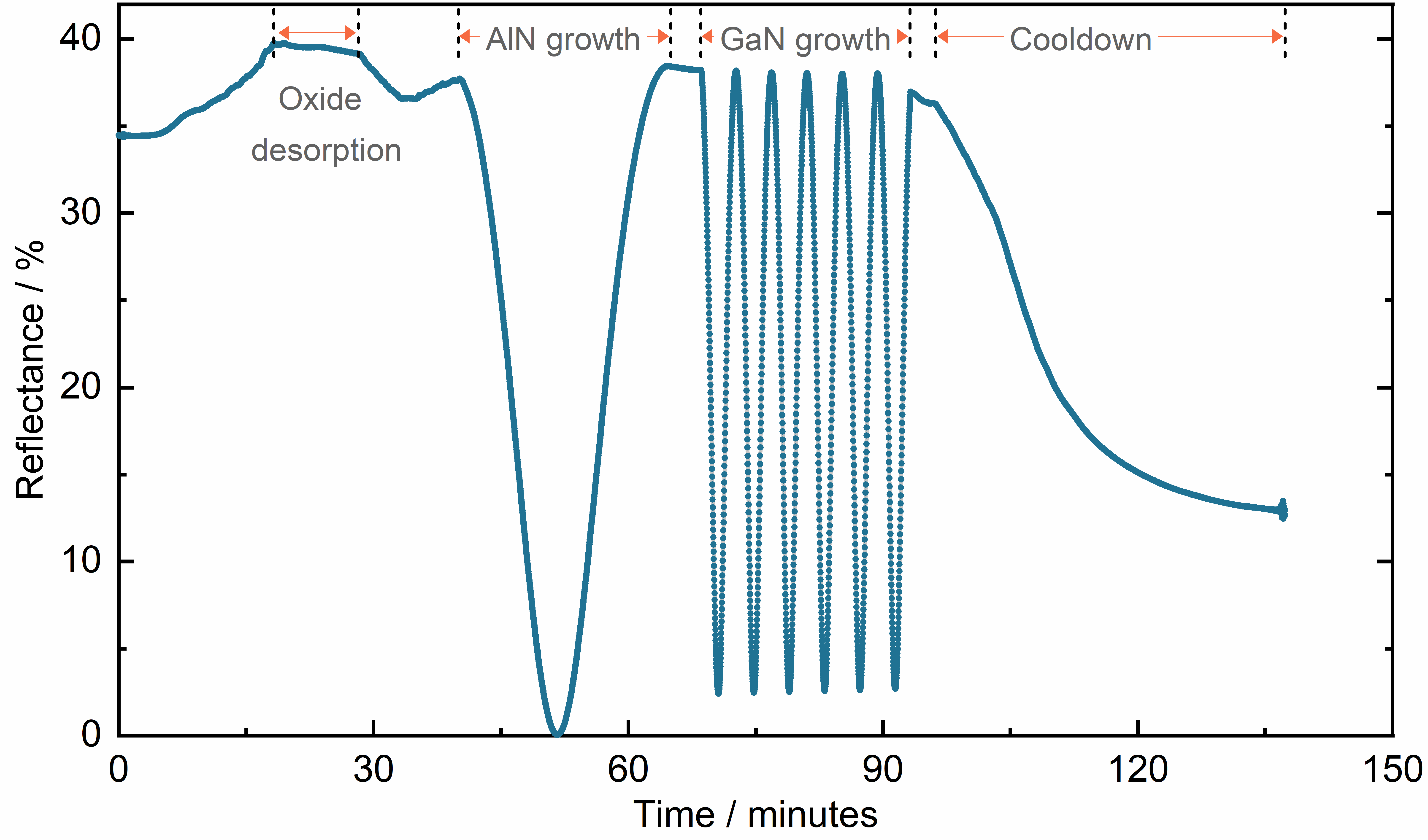


Figure S3 shows the evolution of reflectance during a representative growth run of 800 nm GaN/ 150 nm AlN on a silicon (111) wafer. For this sample, the GaN layer was grown at 75 Torr. The duration of *in situ* oxide desorption, AlN growth (the second stage i.e. ≈ 140 nm thick part), GaN growth, and post-growth cooldown steps are marked. The silicon wafer is loaded directly into the growth chamber without any chemical or thermal treatment beforehand. As seen, after loading (t = 0 s), this entire growth run is completed within 2.5 hours. In contrast to GaN, the growth of AlGaN alloy layers suffers from additional gas-phase pre-reactions and much slower growth rates. E.g., of the various step-graded AlGaN buffer designs described in^[1]^, the growth times for the fastest 1.0 μm and 1.7 μm buffer layers themselves were ≈ 2 hours and ≈ 3.3 hours, respectively. On the other hand, AlN/GaN superlattice (SL) buffers require periodic changes in growth conditions for each layer of the SL. From^[2]^, it can be found that growth of 1.4 μm to 1.8 μm buffers require additional growth times of ≈ 1.5 hours. Thus buffer-less structures are considerably beneficial in terms of thermal budget as well as metal-organic precursors and ammonia usage.


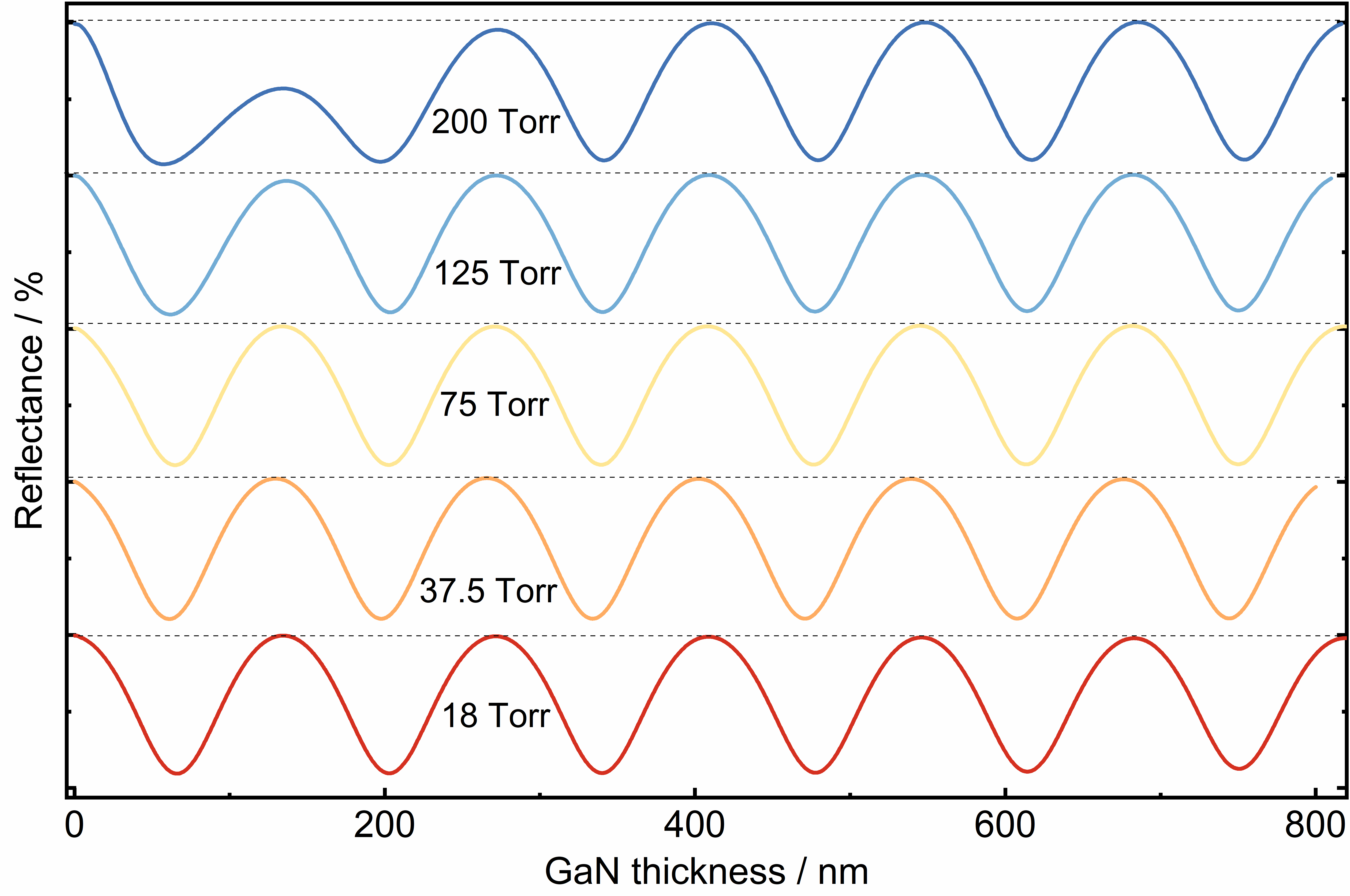


**Supplementary Figure S4**. Variation of real-time reflectance transients with the reactor pressure during the growth of the ≈ 800 nm thick GaN layers.

Figure S4 shows real-time reflectance transients, which were measured during the growth of the ≈ 800 nm thick GaN layers at different reactor pressures. The dashed horizontal line marks the value of peak reflectance of the fifth complete oscillation. For a growth pressures of 75 Torr and below, the reflectance achieves this value by the first complete oscillation peak. However, for higher pressures, increasingly further progression into growth is required. All the oscillations eventually have the same peak-to-peak reflectance. The reflectance at the beginning of the GaN growth was identical for all the growths, and this data has been offset for comparison. For the 650 nm laser wavelength used for these measurements, each complete oscillation corresponds to a thickness of ≈ 137 nm for GaN.


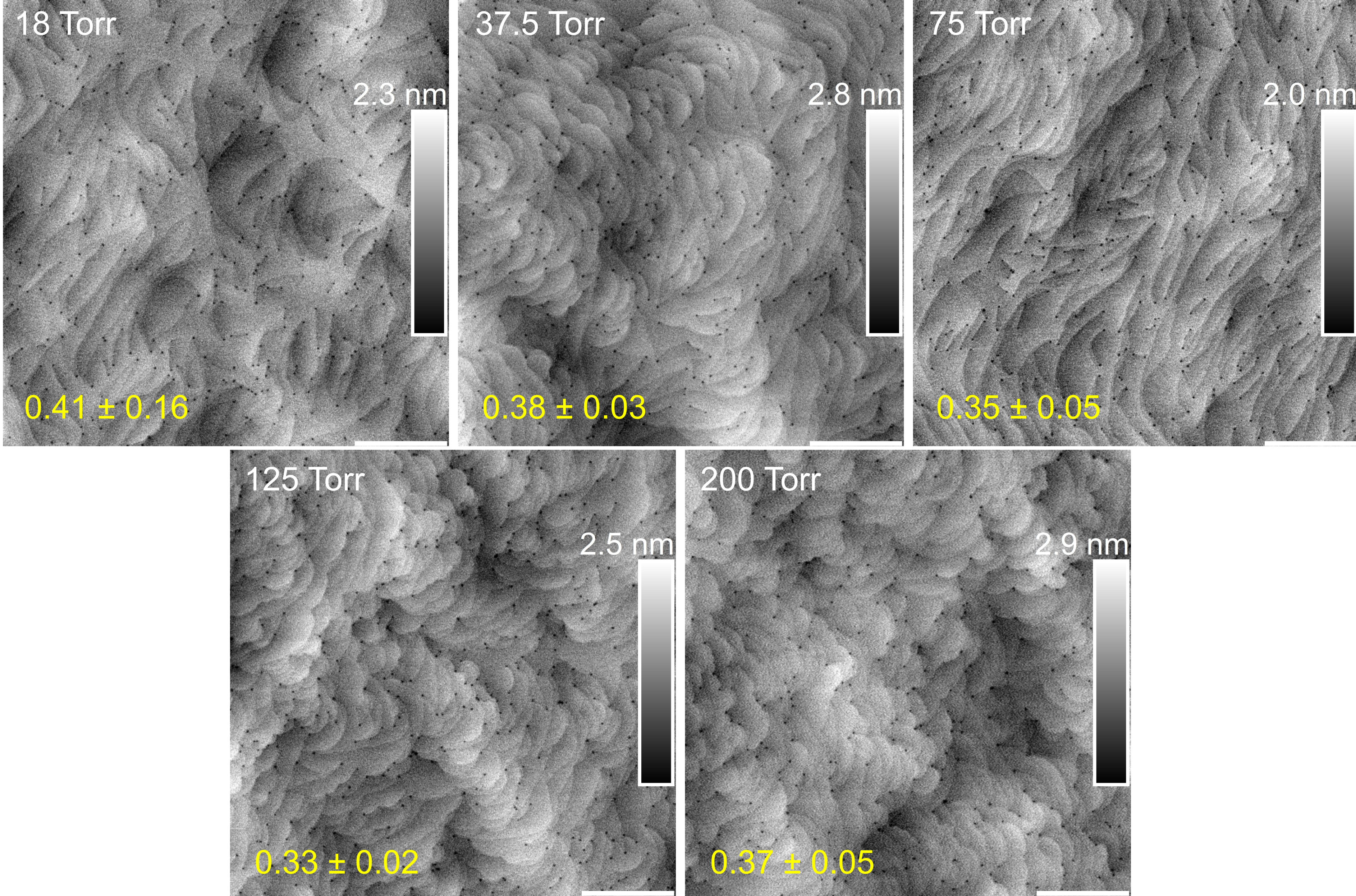


**Supplementary Figure S5**. Topography of the ≈ 800 nm thick GaN layers grown at different pressures. The scale bar in all the 25 µm^2^ images is 1 µm. Root mean square (rms) roughness values in nm are given in yellow on each image.

The topography of the ≈ 800 nm thick GaN layers grown at different pressures is shown in Figure S5. All the 5 µm × 5 µm surfaces show a morphology typical of MOCVD-grown GaN with steps pinned at threading dislocations (which appear as tiny pits in the images). The sub-nm mean rms roughness for all the wafers (annotated on the images) indicates similarly smooth topography regardless of the growth pressure.


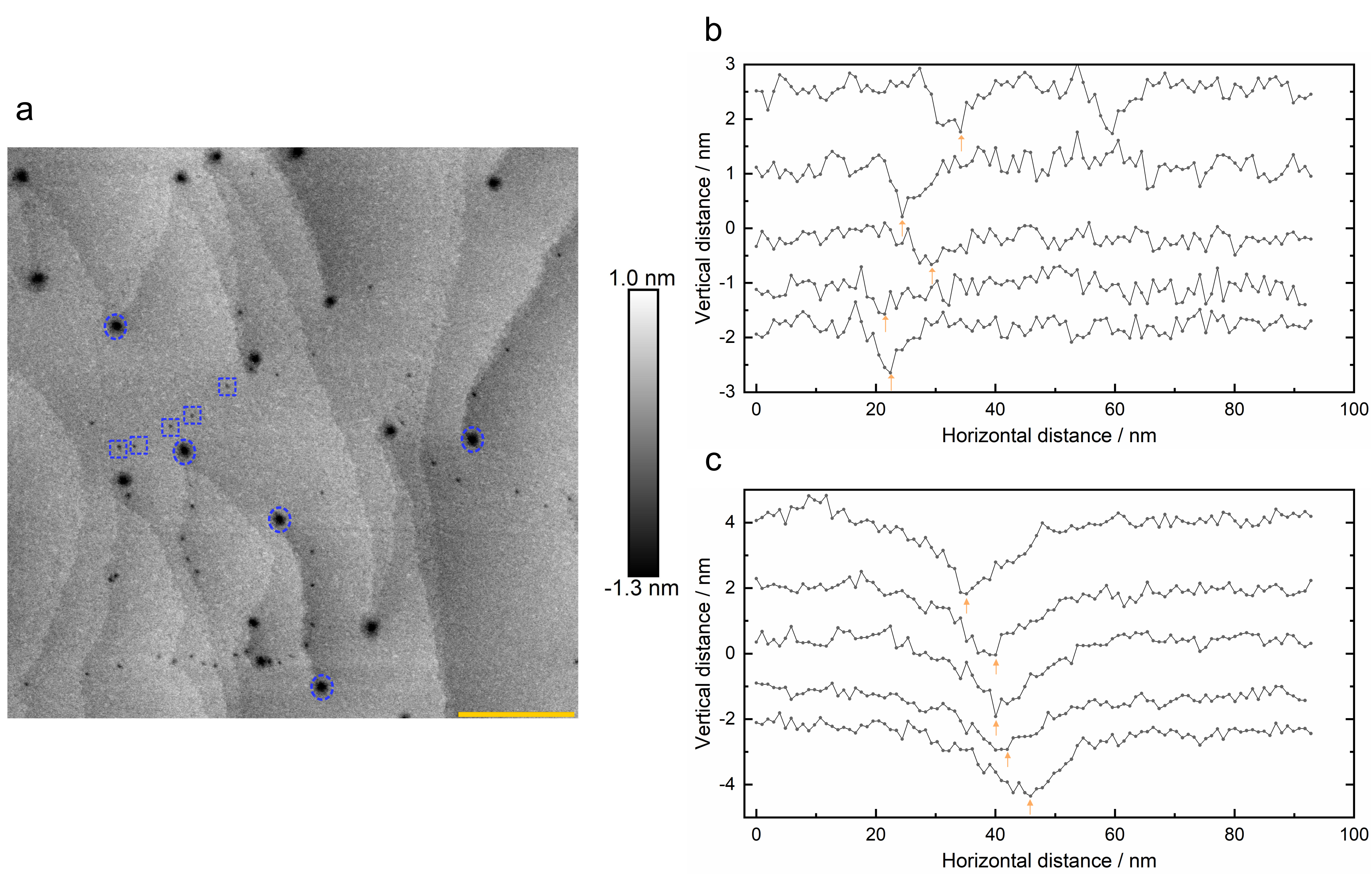


**Supplementary Figure S6**. Dislocation quantification from high-resolution AFM scans of ≈ 800 nm thick GaN samples. (a) A representative 1 μm × 1 μm surface topography of a GaN/AlN/Si wafer and (b)/(c) waterfall plot of line profiles extracted from the AFM dataset. The scale bar in (a) is 200 nm. The arrows in (b) and (c) denotes the maximum measured depth for each profile.

For the dislocation quantification of the ≈ 800 nm thick GaN samples, high-resolution AFM scans have been recorded. Figure S6 (a) shows such a representative 1 µm × 1 µm AFM image of the surface topography of a GaN/AlN/Si wafer in which the GaN layer was grown at 37.5 Torr (scale bar: 200 nm). For each investigated wafer, nine such random areas were scanned for statistics. All the images were acquired with high sampling density to identify the small pits created from surface terminations of threading dislocations. The observed pits created due to dislocations could be categorized as either shallower and narrower or deeper and wider. These were assigned to have either edge or screw characters, respectively. Five of such dislocations with edge character are marked with open squares in (a) and their line profiles are shown in (b). Also, the line profiles five dislocations with screw characters (marked with open circles in (a)) are shown in (c).

Figure S7 shows 3-D FEM (Ansys) thermal simulations of 10 × 350 µm, 60 µm gate pitch AlGaN/GaN HEMTs on 675 µm-thick silicon substrates with (a) conventional buffer and (b) buffer-less design. For the simulations, a power dissipation of 5 W/mm within an 0.5 µm-long region at the drain edge of each gate is considered. The bottom surface of the substrate (not shown) is fixed at 22°C. (a) shows the temperature gradient around the channel heat source in the middle of the central gate finger where the layers between GaN and substrate is replaced with a strain relief layer (SRL) with a TBR_eff_ equivalent to 109 m^2^K/GW.^[3]^ (b) shows our buffer-less design with a 150 nm-thick nucleation layer (as evaluated, TBR_eff_ = 11 m^2^K/GW). As both plots use the same temperature scale for comparison, it is evident that the temperatures in the buffer-less structure throughout are substantially lower. For example, the ΔT across the SRL and nucleation layers shown in (a) and (b) are 56 °C and 15 °C, respectively.


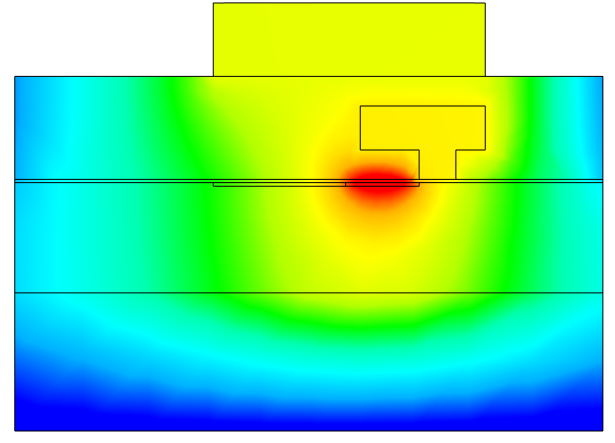


**750nm GaN**

**940 nm SRL**

**Source field plate**

**SiNx**

**Heat source**

**AlGaN**

**Drain**

**Source**

**Gate**


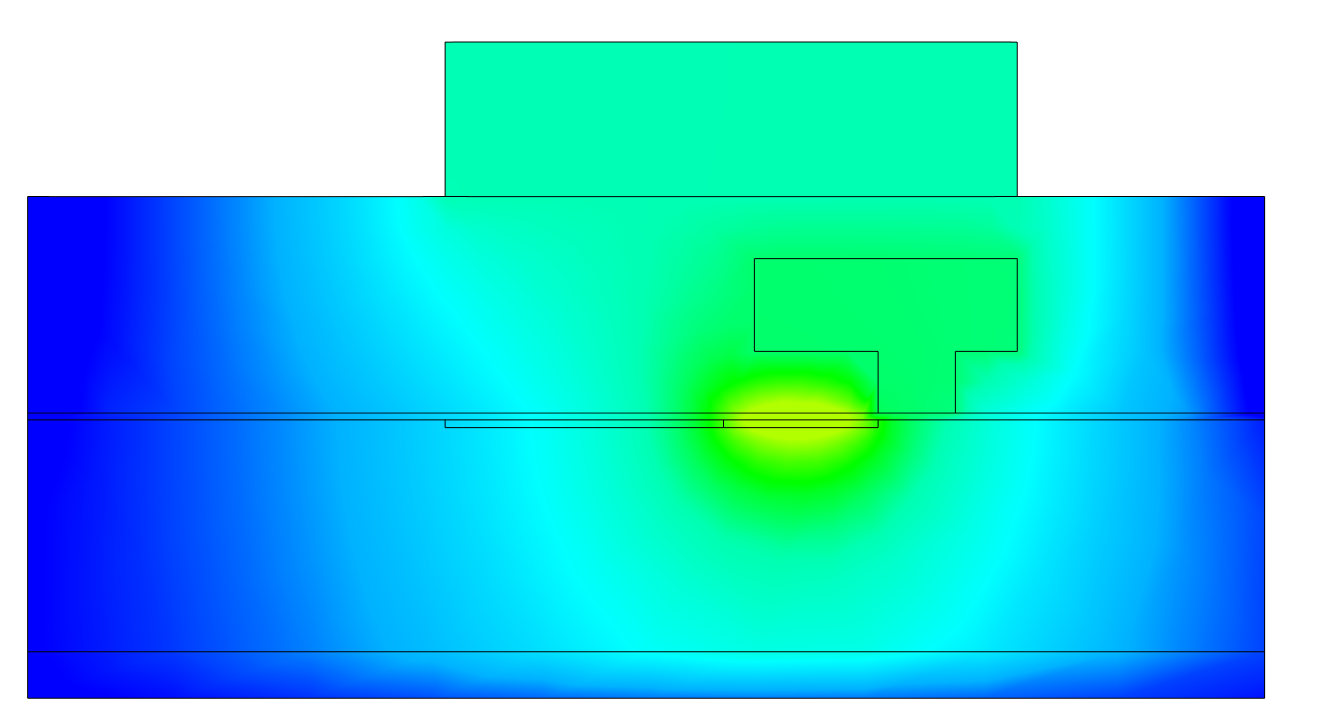


**750nm GaN**

**150nm Nucleation layer**

**Source field plate**

**SiNx**

**Heat source**

**AlGaN**

**Drain**

**Source**

**Gate**


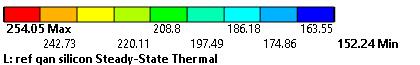


168

254

Temperature (°C)

a

b

**Supplementary Figure S7**. 3-D FEM thermal simulations of 10×350 μm, 60 µm gate pitch AlGaN/GaN HEMTs on 675 µm-thick silicon substrates. (a) Conventional buffer design and (b) buffer-less design.

**Supplementary Figure S8**. XRD reciprocal space maps around the AlN 20-25 reflection for Al_x_Ga_1-x_N/GaN/AlN heterostructure samples with different AlN mole fractions (x = 0.19, 0.22, and 0.26).


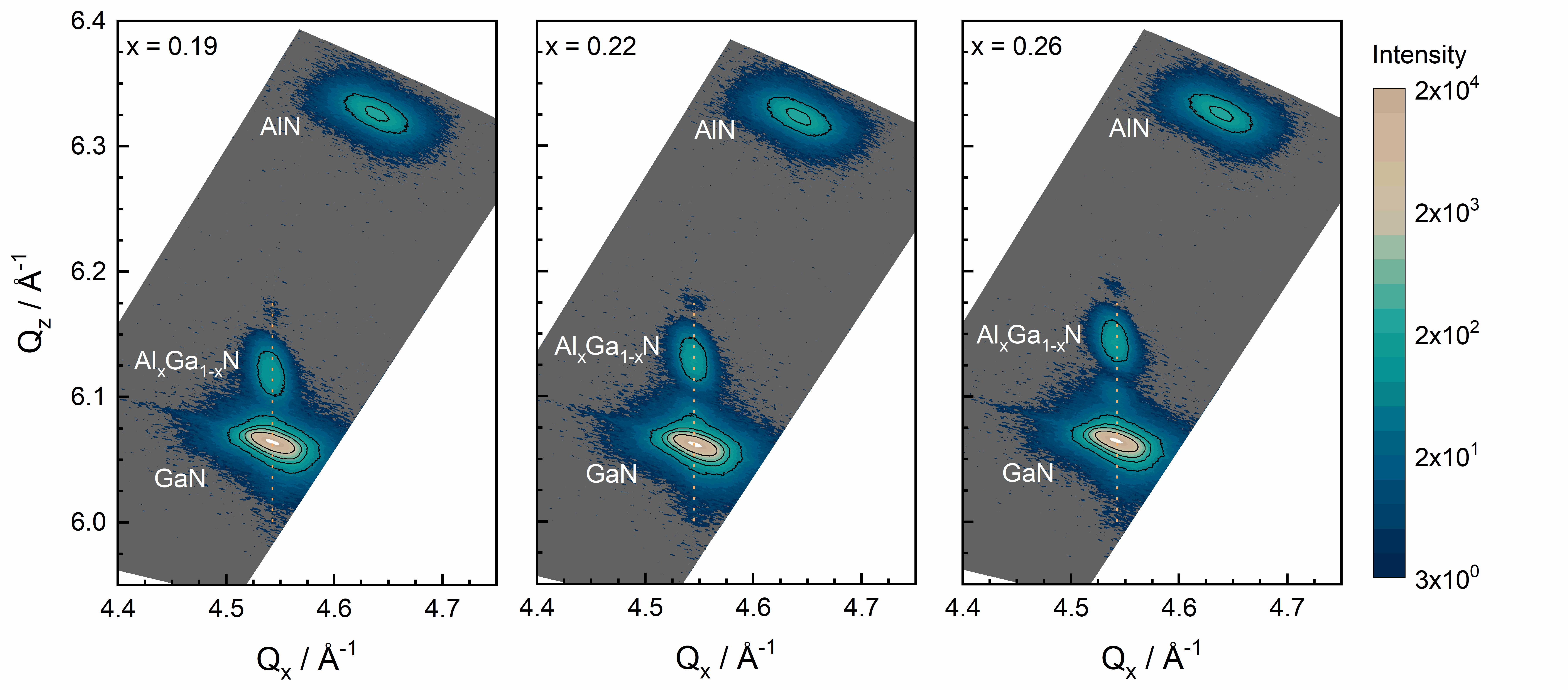


Figure S8 shows XRD reciprocal space maps around the AlN 20-25 reflection for Al_x_Ga_1‑x_N/GaN/AlN heterostructure samples with different AlN mole fractions (x = 0.19, 0.22, and 0.26). For all compositions, the centroid of the reciprocal lattice spot of the barrier layer is vertically aligned with the GaN layer (indicated by dashed lines). Also, as x increases, the AlGaN reflection progressively moves to larger Q_z_ values. These features confirm that for all the studied Al_x_Ga_1‑x_N compositions, the barriers are pseudomorphically strained to the GaN layers underneath.


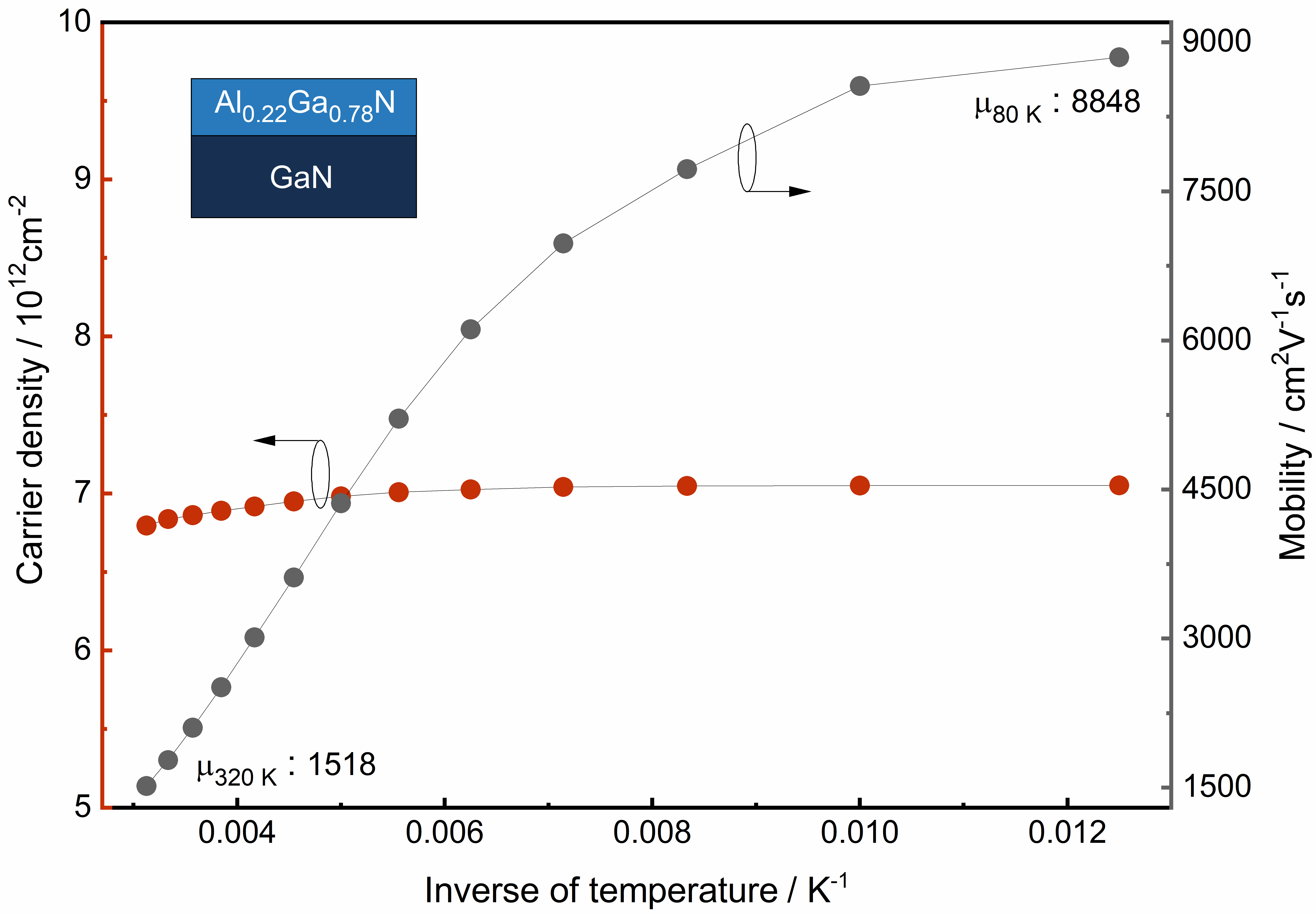


**Supplementary Figure S9**. Temperature-dependent carrier density and Hall-mobility for a sample with Al_0.22_Ga_0.78_N barrier.

Figure S9 shows the carrier density and Hall-mobility for a sample with Al_0.22_Ga_0.78_N barrier as function of inverse temperature. Between 0.003K^-1^ (320 K, n_s_: 6.8×10^12^ cm^-2^) and 0.0125 K^-1^ (80 K, n_s_: 7.1×10^12^ cm^-2^), the carrier density stays nearly constant. In contrast, the mobility continuously rises with a reduction in temperature and reaches 8848 cm^2^V^-1^s^-1^ at 80 K for this sample. This effectively caused a ≈ 6× reduction in the sheet resistance from 605 to 100 Ω/ (not shown). The trendlines are only for a guide to the eye.


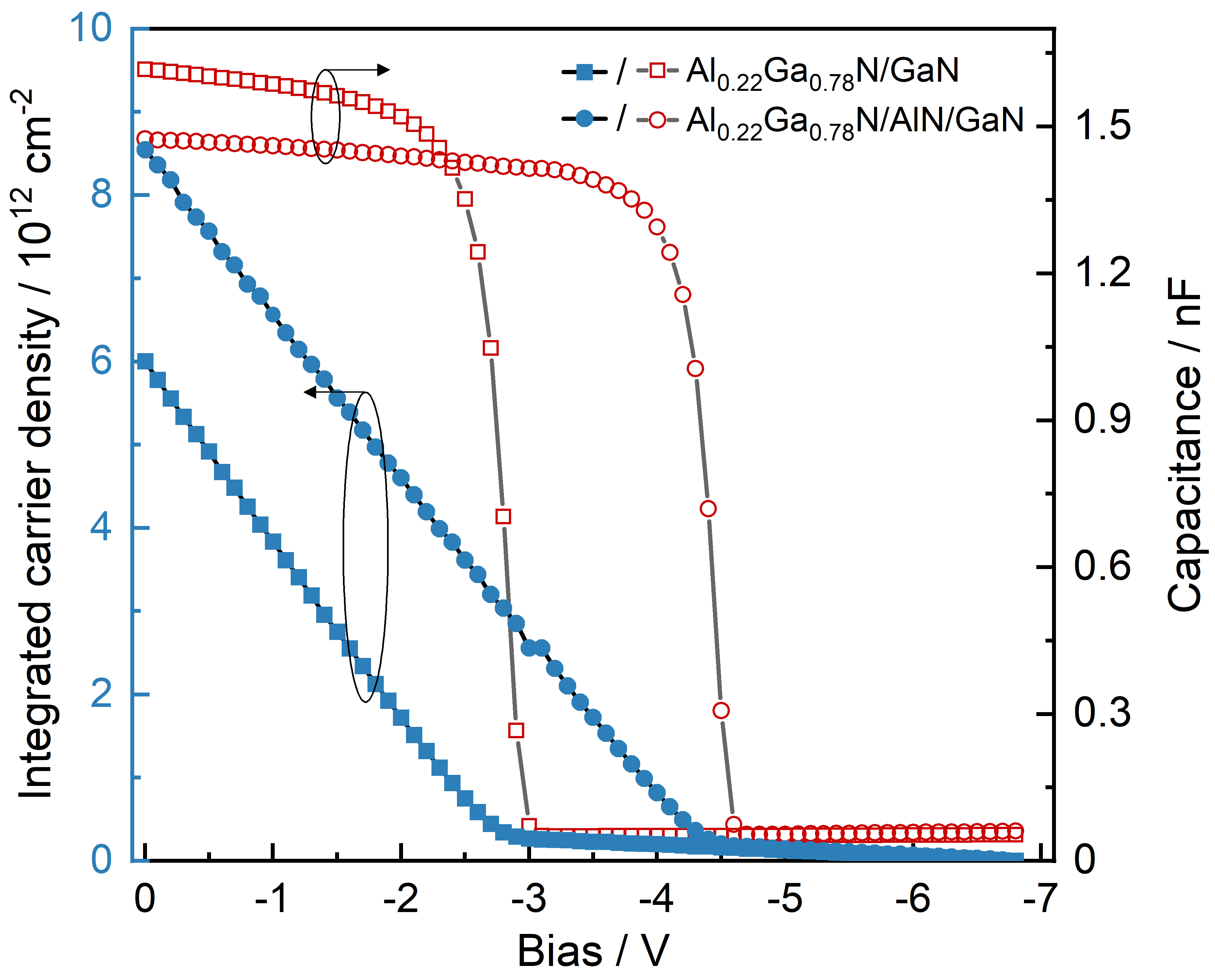

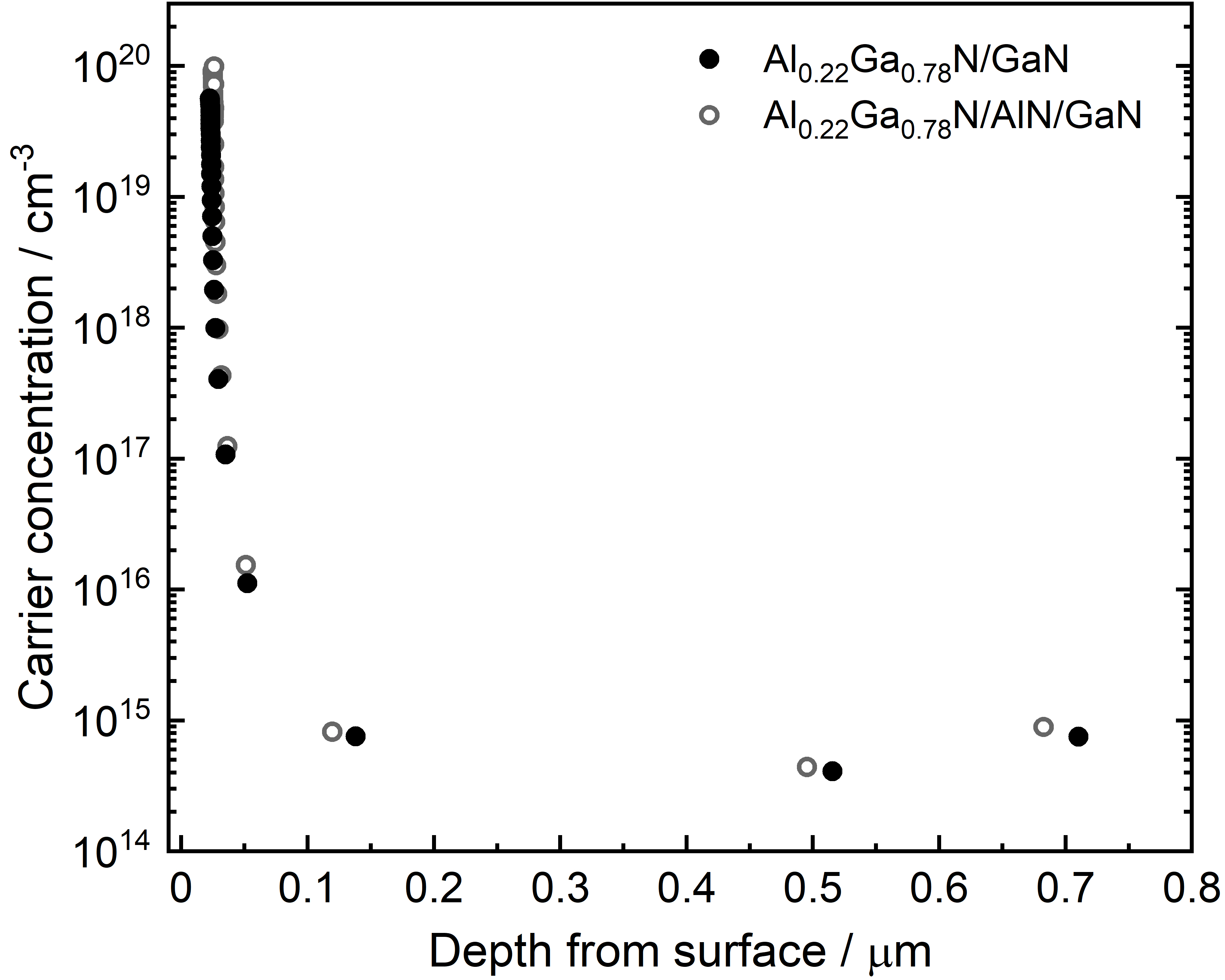
Hg-CV data confirms the 2DEG nature of the carriers for representative Al_0.22_Ga_0.78_N/GaN heterostructure and also shows the effect of adding the AlN spacer. As shown in (a), both samples have an almost invariant capacitance with reverse-bias until the pinch-off voltage (V_p_) when the entire 2DEG gets depleted. V_p_ with and without the AlN layer are -4.3 V and -2.7 V, respectively. The extracted carrier densities are marginally lower than those derived from Hall-effect measurements for these two heterostructures (average values were 8.5×10^12^ /cm^2^ and 6.9×10^12^ /cm^2^, respectively) which is very likely due to the different surface barrier heights for the two different techniques. (b) Further shows that the majority of the carriers are at the interface and the sample with the AlN spacer has a higher peak carrier concentration. Also, inside the GaN layer the carrier concentration is very low (≈ 10^15^ cm^-3^) even without any deep-level doping which attests the high-quality of this layer.

**Supplementary Figure S10**. (a) Capacitance (right y-axis) versus voltage data for Al_0.22_Ga_0.78_N/GaN heterostructures with and without the AlN spacer layer. The extracted carrier density (left y-axis) versus voltage is also shown in the same plot. (b) The variation of carrier concentration across depth for the same samples is derived from the capacitance versus voltage data in (a). The measurements were done in a mercury-probe CV set-up (Hg-CV, MDC make) in a front-front configuration at 10 kHz and ≈ 760 µm diameter of the inner Schottky contact. Note that for (b), due to the very low carrier concentration in the intrinsic GaN layer, the depletion region already extends to the p-type Si at 0.7 µm depth and further data points are not shown.

**a**

**b**


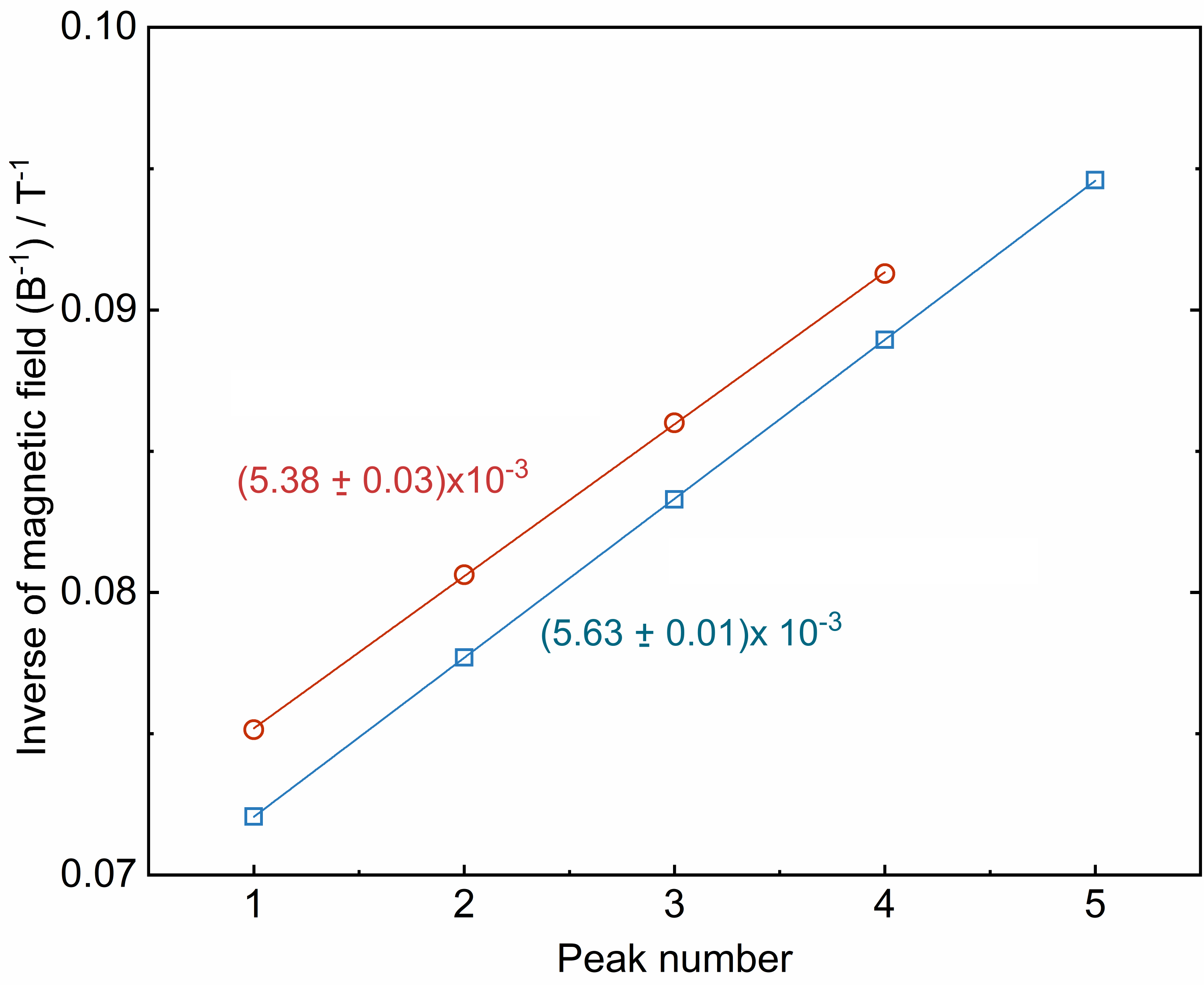


**Supplementary Figure S11**. Position of the successive peaks for the two distinct oscillations observed in the magneto-transport data above 10 T at 1.8 K.

The low-temperature (1.8 K) magneto-transport data presented in the main manuscript show single peaks for the oscillations below 10 T and double peaks for the oscillations above 10T (compare Fig. 6 (b) in the main manuscript). Figure S10 above shows the position of these two successive peaks for the distinct oscillations observed above 10 T. The straight lines are linear fits to the experimental data (open symbols). The extracted slopes (ΔB^-1^) along with their standard error are annotated for each oscillation. For SdH oscillations of carriers in a single sub-band, the carrier density is given by (2e×ΔB^-1^/h). As these oscillations arise due to the spin splitting of the same sub-band, the formula for the associated carriers should be (e×ΔB^-1^/h) i.e. without the spin degeneracy factor of 2. This yields spin-up (n↑) and spin-down (n↓) densities ≈ 4.47×10^12^ cm^-2^ and ≈ 4.31×10^12^ cm^-2^, respectively. ^123^

**Table S1**. Summary of effective thermal boundary resistances (TBR_eff_) between heteroepitaxial GaN and the various non-native substrates plotted in Fig. 4d of the main manuscript. Sample ID refers to the symbol used for that article in the main text and reference denotes the reference number in this supplementary material. The interlayer type and its thicknesses are also listed if this information is available.

| Substrate | Sample-ID [Reference] | Interlayer  type | Thick-  ness / nm | TBR_eff_ /  m^2^ K GW^-1^ | Note |
| --- | --- | --- | --- | --- | --- |
| Sapphire | S1^4^[4] | AlN | 40 | 300 | The mean value was plotted from upper and lower bounds. |
| Sapphire | S2^5^[5] | - | 30 | 120 | The exact type was interlayer was not mentioned. |
| SiC | S2^5^[5] | - | 30 | 33 | The exact type was interlayer was not mentioned. |
| SiC | S3^3^[3] | AlN | 30 | 14 | Value was extracted from a figure in the article. |
| SiC | S4^6^[6] | AlN | 40 | 120 | Thickness was not explicitly mentioned. We assumed it to be 40 nm. |
| SiC | S5^7^[7] | AlN | 90 | 25 | - |
| SiC | S5^7^[7] | 24% AlGaN | 3 | 20 | Not shown in our plot. |
| SiC | S6^8^[8] | AlN | 40 | 36.8 | Value was extracted from a figure in the article. In the article, TBR_eff_ was shown to increase with temperature. We plotted the value for the lowest temperature. |
| SiC | S6^8^[8] | AlN | 80 | 25.8 | Same as above. |
| SiC | S6^8^[8] | AlN | 30 | 35 | Same as above. |
| SiC | S7^9^[9] | AlN | 40 | 8.4 | Same as above. |
| SiC | S7^9^[9] | AlN | 70 | 20 | Same as above. |
| SiC | S7^9^[9] | AlN | 70 | 24.2 | Same as above. |
| Si | S2^5^[5] | AlN and strain-relief layer | 1000 | 33 | The exact type of the strain-relief layer was not mentioned. As its thickness was also not given, we assumed it to be 1000 nm. |
| Si | S3^3^[3] | Several layers of AlGaN | 940 | 109.3 | This plotted value is only for the buffer. Including the NL and the associated interfaces, total thermal resistance would be higher. The compositions of the AlGaN were not specified. |
| Si | S4^6^[6] | LT AlN +GaN+  LT AlN | 487 | 70 | - |
| Si | S8^10^[10] | AlN/GaN SL only | 780 | 112 | - |
| Si | S8^10^[10] | AlN | 100 | 5.3 | - |
| Si | S8^10^[10] | AlN | 100 | 7 | - |
| Si | This work | AlN | 150 | 11 | - |

**Table S2**. Electron density (n_s_) and mobility (μ) of the various AlGaN/GaN-based high electron mobility 2DEGs plotted in Fig. 5d of the main text. For consistency, only Hall-effect measured values at room-temperature are considered for comparison. The specification of the MOCVD-grown epilayers on Si(111) for each sample is also noted.

| Sample-ID [Reference] | Wafer  dia. / inch | t_AlN_  / nm | Buffer type  [Thickness / nm] | t_GaN_  / nm | Total epi.*  / nm | n_s_  / 10^12^ cm^-2^ | μ /  cm^2^⋅V^-1^⋅s^-1^ |
| --- | --- | --- | --- | --- | --- | --- | --- |
| S9^11^[11] | 6 | 150 | Graded AlGaN [1700] | 2750 | 4600 | 7.2 | 2250 |
| S10^12^[12] | 4 | 284 | Graded AlGaN [1155] | 1250 | 2689 | 7.2 | 2161 |
| S11^13^[13] | 4 | 270 | AlGaN single layer [330] | 2000 | 2600 | 9.3 | 2150 |
| S12^14^[14] | 4 | 270 | AlGaN single layer [330] | 3000 | 3600 | 8.4 | 2040 |
| S13^15^[15] | 8 | NA | AlGaN/AlN  multilayer [NA] | NA | 5000 | 7.8** | 2000 |
| S14^16^[16] | 6 | NA | NA [NA] | NA | 4500 | 9.7 | 1970 |
| S15^17^[17] | 6 | 100 | Graded AlGaN [600] | 500 | 1200 | 6.9 | 1898 |
| S16^17^[17] | 6 | 100 | Graded AlGaN  [1000] | 500 | 1600 | 8.0 | 1893 |
| S17^18^[18] | 6 | 193 | Graded AlGaN  [976] | 1260 | 2429 | 10.0 | 1800 |
| S18^19^[19] | NA | NA | NA [2000] | 6000 | 8000 | 8.9 | 1760 |
| S19^20^[20] | 8 | 150 | AlGaN single layer with superlattice [2050] | 1000 | 3200 | 11.6 | 1720 |
| S20^21^[21] | 2 | 200 | Buffer-less | 300 | 500 | 8.1^#^ | 1700 |
| S21^17^[17] | 6 | 100 | Graded AlGaN  [1000] | 500 | 1600 | 7.6 | 1676 |
| S22^22^[22] | 4 | 180 | AlGaN single layer [2300] | 150 | 2630 | 12.0^##^ | 1670^##^ |
| S23^23^[23] | NA | NA | AlGaN based buffer [4500] | 200 | 4700 | 7.6 | 1620 |
| S24^24^[24] | 8 | 130 | Buffer-less | 950 | 1080 | 9.2 | 1593 |
| S25^25^[25] | 4 | 100 | AlGaN single layer with Superlattice  [ ≥ 4000] | ≥ 500 | 5500 | 9.6** | 1423 |
| S26^26^[26] | 4 | 80 | AlGaN single layer with Superlattice [3030] | 1000 | 4110 | 7.6 | 1350 |
| S27^27^[27] | 2 | 100 | Graded AlGaN  [1000] | 500 | 1600 | 8.4 | 1325 |
| S28^28^[28] | 2 | 200 | Buffer-less | 500 | 700 | 9.1 | 1320 |
| This work | 6 | 150 | Buffer-less | 800 | 950 | 8.5 | 2014 |
| This work | 6 | 150 | Buffer-less | 800 | 950 | 9.6 | 1849 |

*Total epilayer thickness excludes the barrier thickness (usually ≈ 20 nm)

*^#^* Same sample before passivation showed ns to be 4.9×10^12^ cm^-2^

*n_s_ not given, calculated from R_sheet_ and μ

*^# #^*after substrate removal

References

(1) Ghosh, S.; Hinz, A. M.; Frentrup, M.; Alam, S.; Wallis, D. J.; Oliver, R. A. Design of Step-Graded AlGaN Buffers for GaN-on-Si Heterostructures Grown by MOCVD. *Semicond. Sci. Technol.* **2023**, *38* (4), 044001. https://doi.org/10.1088/1361-6641/acb9b6.

(2) Lin, P.-J.; Huang, S.-Y.; Wang, W.-K.; Chen, C.-L.; Chung, B.-C.; Wuu, D.-S. Controlling the Stress of Growing GaN on 150-Mm Si (111) in an AlN/GaN Strained Layer Superlattice. *Appl. Surf. Sci.* **2016**, *362*, 434–440. https://doi.org/10.1016/j.apsusc.2015.11.226.

(3) Yuan, C.; Pomeroy, J. W.; Kuball, M. Above Bandgap Thermoreflectance for Non-Invasive Thermal Characterization of GaN-Based Wafers. *Appl. Phys. Lett.* **2018**, *113* (10). https://doi.org/10.1063/1.5040100.

(4) Turin, V. O.; Balandin, A. A. Performance Degradation of GaN Field-Effect Transistors Due to Thermal Boundary Resistance at GaN∕substrate Interface. *Electron. Lett.* **2004**, *40* (1), 81. https://doi.org/10.1049/el:20040071.

(5) Sarua, A.; Ji, H.; Hilton, K. P.; Wallis, D. J.; Uren, M. J.; Martin, T.; Kuball, M. Thermal Boundary Resistance Between GaN and Substrate in AlGaN/GaN Electronic Devices. *IEEE Trans. Electron Devices* **2007**, *54* (12), 3152–3158. https://doi.org/10.1109/TED.2007.908874.

(6) Kuzmík, J.; Bychikhin, S.; Pogany, D.; Gaquière, C.; Pichonat, E.; Morvan, E. Investigation of the Thermal Boundary Resistance at the III-Nitride/Substrate Interface Using Optical Methods. *J. Appl. Phys.* **2007**, *101* (5). https://doi.org/10.1063/1.2435799.

(7) Feng, Y.; Sun, H.; Yang, X.; Liu, K.; Zhang, J.; Shen, J.; Liu, D.; Cai, Z.; Xu, F.; Tang, N.; Yu, T.; Wang, X.; Ge, W.; Shen, B. High Quality GaN-on-SiC with Low Thermal Boundary Resistance by Employing an Ultrathin AlGaN Buffer Layer. *Appl. Phys. Lett.* **2021**, *118* (5). https://doi.org/10.1063/5.0037796.

(8) Riedel, G. J.; Pomeroy, J. W.; Hilton, K. P.; Maclean, J. O.; Wallis, D. J.; Uren, M. J.; Martin, T.; Forsberg, U.; Lundskog, A.; Kakanakova-Georgieva, A.; Pozina, G.; Janzen, E.; Lossy, R.; Pazirandeh, R.; Brunner, F.; Wurfl, J.; Kuball, M. Reducing Thermal Resistance of AlGaN/GaN Electronic Devices Using Novel Nucleation Layers. *IEEE Electron Device Lett.* **2009**, *30* (2), 103–106. https://doi.org/10.1109/LED.2008.2010340.

(9) Manoi, A.; Pomeroy, J. W.; Killat, N.; Kuball, M. Benchmarking of Thermal Boundary Resistance in AlGaN/GaN HEMTs on SiC Substrates: Implications of the Nucleation Layer Microstructure. *IEEE Electron Device Lett.* **2010**, *31* (12), 1395–1397. https://doi.org/10.1109/LED.2010.2077730.

(10) Bougher, T. L.; Yates, L.; Lo, C.-F.; Johnson, W.; Graham, S.; Cola, B. A. Thermal Boundary Resistance in GaN Films Measured by Time Domain Thermoreflectance with Robust Monte Carlo Uncertainty Estimation. *Nanoscale Microscale Thermophys. Eng.* **2016**, *20* (1), 22–32. https://doi.org/10.1080/15567265.2016.1154630.

(11) Heuken, L.; Alshahed, M.; Ottaviani, A.; Alomari, M.; Fahle, D.; Heuken, M.; Burghartz, J. N. Temperature Dependent Lateral and Vertical Conduction Mechanisms in AlGaN/GaN HEMT on Thinned Silicon Substrate. *Jpn. J. Appl. Phys.* **2019**, *58* (SC), SCCD11. https://doi.org/10.7567/1347-4065/ab0406.

(12) Xu, X.; Zhong, J.; So, H.; Norvilas, A.; Sommerhalter, C.; Senesky, D. G.; Tang, M. Wafer-Level MOCVD Growth of AlGaN/GaN-on-Si HEMT Structures with Ultra-High Room Temperature 2DEG Mobility. *AIP Adv.* **2016**, *6* (11). https://doi.org/10.1063/1.4967816.

(13) Cheng, J.; Yang, X.; Sang, L.; Guo, L.; Zhang, J.; Wang, J.; He, C.; Zhang, L.; Wang, M.; Xu, F.; Tang, N.; Qin, Z.; Wang, X.; Shen, B. Growth of High Quality and Uniformity AlGaN/GaN Heterostructures on Si Substrates Using a Single AlGaN Layer with Low Al Composition. *Sci. Rep.* **2016**, *6* (1), 23020. https://doi.org/10.1038/srep23020.

(14) Cheng, J.; Yang, X.; Sang, L.; Guo, L.; Hu, A.; Xu, F.; Tang, N.; Wang, X.; Shen, B. High Mobility AlGaN/GaN Heterostructures Grown on Si Substrates Using a Large Lattice-Mismatch Induced Stress Control Technology. *Appl. Phys. Lett.* **2015**, *106* (14). https://doi.org/10.1063/1.4917504.

(15) Ikejiri, K.; Hiroyama, Y.; Kasahara, K.; Hirooka, C.; Osada, T.; Tanaka, M.; Takada, T.; Egawa, T. Mass Production-Ready Characteristics of AlGaN/AlN/GaN High-Electron-Mobility Transistor Structures Grown on 200 Mm Diameter Silicon Substrates Using Metal-Organic Chemical Vapor Deposition. *Semicond. Sci. Technol.* **2021**, *36* (1), 014004. https://doi.org/10.1088/1361-6641/abc3da.

(16) Mohanty, S. K.; Chen, Y.-Y.; Yeh, P.-H.; Horng, R.-H. Thermal Management of GaN-on-Si High Electron Mobility Transistor by Copper Filled Micro-Trench Structure. *Sci. Rep.* **2019**, *9* (1), 19691. https://doi.org/10.1038/s41598-019-56292-3.

(17) Mohan, N.; Manikant; Soman, R.; Raghavan, S. Integrating AlGaN/GaN High Electron Mobility Transistor with Si: A Comparative Study of Integration Schemes. *J. Appl. Phys.* **2015**, *118* (13). https://doi.org/10.1063/1.4932148.

(18) Liu, H. F.; Dolmanan, S. B.; Zhang, L.; Chua, S. J.; Chi, D. Z.; Heuken, M.; Tripathy, S. Influence of Stress on Structural Properties of AlGaN/GaN High Electron Mobility Transistor Layers Grown on 150 Mm Diameter Si (111) Substrate. *J. Appl. Phys.* **2013**, *113* (2). https://doi.org/10.1063/1.4774288.

(19) Kagawa, R.; Kawamura, K.; Sakaida, Y.; Ouchi, S.; Uratani, H.; Shimizu, Y.; Ohno, Y.; Nagai, Y.; Liang, J.; Shigekawa, N. AlGaN/GaN/3C-SiC on Diamond HEMTs with Thick Nitride Layers Prepared by Bonding-First Process. *Appl. Phys. Express* **2022**, *15* (4), 041003. https://doi.org/10.35848/1882-0786/ac5ba7.

(20) Yamaoka, Y.; Kakamu, K.; Ubukata, A.; Yano, Y.; Tabuchi, T.; Matsumoto, K.; Egawa, T. Influence of the Al Content of the AlGaN Buffer Layer in AlGaN/GaN High‐electron‐mobility Transistor Structures on a Si Substrate. *Phys. status solidi* **2017**, *214* (3). https://doi.org/10.1002/pssa.201600618.

(21) Frayssinet, E.; Leclaire, P.; Mohdad, J.; Latrach, S.; Chenot, S.; Nemoz, M.; Damilano, B.; Cordier, Y. Influence of Metal-Organic Vapor Phase Epitaxy Parameters and Si(111) Substrate Type on the Properties of AlGaN/GaN HEMTs with Thin Simple Buffer. *Phys. status solidi* **2017**, *214* (4), 1600419. https://doi.org/10.1002/pssa.201600419.

(22) Srivastava, P.; Das, J.; Visalli, D.; Derluyn, J.; Van Hove, M.; Malinowski, P. E.; Marcon, D.; Geens, K.; Cheng, K.; Degroote, S.; Leys, M.; Germain, M.; Decoutere, S.; Mertens, R. P.; Borghs, G. Silicon Substrate Removal of GaN DHFETs for Enhanced (<1100 V) Breakdown Voltage. *IEEE Electron Device Lett.* **2010**, *31* (8), 851–853. https://doi.org/10.1109/LED.2010.2050673.

(23) Condori Quispe, H. O.; Chanana, A.; Encomendero, J.; Zhu, M.; Trometer, N.; Nahata, A.; Jena, D.; Xing, H. G.; Sensale-Rodriguez, B. Comparison of Unit Cell Coupling for Grating-Gate and High Electron Mobility Transistor Array THz Resonant Absorbers. *J. Appl. Phys.* **2018**, *124* (9). https://doi.org/10.1063/1.5032102.

(24) Lee, H.-P.; Perozek, J.; Rosario, L. D.; Bayram, C. Investigation of AlGaN/GaN High Electron Mobility Transistor Structures on 200-Mm Silicon (111) Substrates Employing Different Buffer Layer Configurations. *Sci. Rep.* **2016**, *6* (1), 37588. https://doi.org/10.1038/srep37588.

(25) Selvaraj, S. L.; Watanabe, A.; Wakejima, A.; Egawa, T. 1.4-KV Breakdown Voltage for AlGaN/GaN High-Electron-Mobility Transistors on Silicon Substrate. *IEEE Electron Device Lett.* **2012**, *33* (10), 1375–1377. https://doi.org/10.1109/LED.2012.2207367.

(26) Christy, D.; Watanabe, A.; Egawa, T. Influence of Strain Induced by AlN Nucleation Layer on the Electrical Properties of AlGaN/GaN Heterostructures on Si(111) Substrate. *AIP Adv.* **2014**, *4* (10). https://doi.org/10.1063/1.4897338.

(27) Chandrasekar, H.; Mohan, N.; Bardhan, A.; Bhat, K. N.; Bhat, N.; Ravishankar, N.; Raghavan, S. An Early In-Situ Stress Signature of the AlN-Si Pre-Growth Interface for Successful Integration of Nitrides with (111) Si. *Appl. Phys. Lett.* **2013**, *103* (21). https://doi.org/10.1063/1.4831968.

(28) Leclaire, P.; Frayssinet, E.; Morelle, C.; Cordier, Y.; Théron, D.; Faucher, M. Piezoelectric MEMS Resonators Based on Ultrathin Epitaxial GaN Heterostructures on Si. *J. Micromechanics Microengineering* **2016**, *26* (10), 105015. https://doi.org/10.1088/0960-1317/26/10/105015.
